# Ultra-long-acting tunable biodegradable and removable controlled release implants for drug delivery

**DOI:** 10.1038/s41467-019-12141-5

**Published:** 2019-09-20

**Authors:** S. Rahima Benhabbour, Martina Kovarova, Clinton Jones, Daijha J. Copeland, Roopali Shrivastava, Michael D. Swanson, Craig Sykes, Phong T. Ho, Mackenzie L. Cottrell, Anush Sridharan, Samantha M. Fix, Orrin Thayer, Julie M. Long, Daria J. Hazuda, Paul A. Dayton, Russell J. Mumper, Angela D. M. Kashuba, J. Victor Garcia

**Affiliations:** 1 UNC_NCSU Joint Department of Biomedical Engineering, University of North Carolina at Chapel Hill, Chapel Hill, NC USA; 2 UNC Eshelman School of Pharmacy, Division of Pharmacoengineering and Molecular Pharmaceutics, Chapel Hill, NC USA; 3 International Center for the Advancement of Translational Science, Division of Infectious Diseases, Center for Aids Research, School of Medicine, University of North Carolina at Chapel Hill, Chapel Hill, NC USA; 4 Division of Pharmacotherapy and Experimental Therapeutics, UNC Eshelman School of Pharmacy, University of North Carolina at Chapel Hill, Chapel Hill, NC USA; 5 Infectious Disease Biology, Merck Research Laboratories, West Point, PA USA; 6 University of Georgia, Office of the Provost, Athens, GA USA

**Keywords:** Biomaterials, Drug delivery, Chemistry, Materials science

## Abstract

Here we report an ultra-long-acting tunable, biodegradable, and removable polymer-based delivery system that offers sustained drug delivery for up to one year for HIV treatment or prophylaxis. This robust formulation offers the ability to integrate multiple drugs in a single injection, which is particularly important to address the potential for drug resistance with monotherapy. Six antiretroviral drugs were selected based on their solubility in *N*-methyl-2-pyrrolidone and relevance as a combination therapy for HIV treatment or prevention. All drugs released with concentrations above their protein-adjusted inhibitory concentration and retained their physical and chemical properties within the formulation and upon release. The versatility of this formulation to integrate multiple drugs and provide sustained plasma concentrations from several weeks to up to one year, combined with its ability to be removed to terminate the treatment if necessary, makes it attractive as a drug delivery platform technology for a wide range of applications.

## Abstract (web summary)

Patient drug regime compliance is a major issue; sustained release implants could address this. Here, the authors report on a phase inverted in situ forming implant of PLGA for the sustained release of antiretroviral drugs and optimize and demonstrate the release of 6 different drugs over a period of up to a year.

## Introduction

In situ forming implants or ISFIs are a class of material systems that have successfully been used for sustained drug delivery^1–13^. ISFI formulations offer great flexibility in drug delivery, with the capacity to achieve release profiles ranging from days to months^8,14^. These technologies are based on a liquid system centering on the use of a biocompatible, water-miscible organic solvent for the co-dissolution and delivery of both the drug and a biodegradable polymer or other release rate-limiting additive(s)^15–17^. Following injection into the intramuscular or subcutaneous space, the water-miscible solvent diffuses from the mixture into the surrounding space. As the concentration of the organic solvent is depleted at the injection site, the solution undergoes a phase inversion forming a solid implant comprised of the drug and biodegradable polymer^4,8,18,19^. NMP has seen frequent application as the solvent component in ISFIs because of its versatility to solubilize both drugs and polymers, and because of its acceptable safety profile at controlled doses^20–22^. Biodegradable ISFIs do not require surgical removal once the drug is completely released from the implant since these depots slowly degrade into non-toxic byproducts in the body. Specifically, poly(DL-lactide-co-glycolide) (PLGA) is highly soluble in NMP and is biocompatible and biodegradable-eventually degrading to produce glycolic acid and lactic acid monomers-making it a popular choice as a rate-controlling additive^23^. This is not the case for non-biodegradable solid implants, which do require surgical removal once the drug is depleted. PLGA also allows for extensive fine-tuning of drug release kinetics through adjustment of polymer concentration and careful selection from the wide variety of lactic/glycolic acid (L/G) ratios and molecular weights (MW) that are commercially available^15,24^.

ISFI systems exhibit phase inversion upon injection into a subcutaneous or intramuscular environment. These polymeric implants exhibit release profiles that have three distinct phases, a burst release phase that occurs within the first 24–48 h whereby drug is released rapidly from the implant^25^. The other two phases are generated by diffusion-mediated and polymer degradation-mediated release of drug from the implant. The release kinetics in each phase can be fine-tuned by modifying the ratio of polymer to solvent, the polymer properties (MW, backbone, end-group), or by use of a co-solvent or rate-controlling additives in the formulation^25–30^. Other factors that control the release kinetics of a drug from an ISFI system include drug physical/chemical properties and drug affinity to the solvent system and/or polymer. In addition, highly solubilizing and water-miscible solvents like NMP lead to rapid phase inversion and the formation of highly porous polymeric networks. Therefore, when designing these ISFI systems for sustained drug delivery, it is important to consider all the aforementioned contributing factors in order to obtain the desired target release kinetics to achieve therapeutic effects.

The goal of this work was to develop a delivery system that can offer ultra-long-acting drug release by (1) providing flexibility in the choice of active ingredient, (2) sustained release for weeks or months, (3) the ability to be surgically removed in case of an allergic or adverse reaction, (4) and the ability to integrate multiple drugs. We investigate fourteen (14) antiretroviral drugs for their suitability to be formulated into ISFIs and demonstrate the ability to formulate six (6) of them into ISFIs individually (single drug ISFI) or in combination with one or two other drugs (combination drug ISFI) and achieve sustained plasma concentrations from one month to up to 1 year.

## Results

### Solubility of ARV drugs in *N*-methyl-2-pyrrolidone (NMP)

Fourteen (14) antiretroviral drugs from different classes were tested for solubility in NMP (Supplementary Table 1). These drugs were selected based on their relevance for HIV treatment or prevention. Out of the fourteen drugs tested in NMP, based on their solubility properties (LogP) six (6) were eventually tested in the ISFI formulation. Both hydrophilic and hydrophobic drugs were included. From the class of integrase inhibitors, we tested MK-2048 and Dolutegravir (DTG). From the class of protease inhibitors (PIs) we tested Darunavir and Atazanavir; and from the class of non-nucleoside reverse transcriptase inhibitors (NNRTIs) we tested Rilpivirine. Ritonavir, a drug often used as a booster for protease inhibitors was also included in some formulations.

From the class of integrase inhibitors, the highest solubility in NMP was recorded for MK-2048, a second-generation integrase inhibitor developed by Bar-Magen et al.^31^. MK-2048 is highly potent against HIV-1 with in vitro 90% inhibitory concentration (IC90) of ~0.033 μg/mL^31–33^. MK-2048 has low solubility in common biocompatible organic solvents (propylene glycol 5.32 mg/mL, PEG 400 15.6 mg/mL, ethanol 11.3 mg/mL) and poor solubility in water (LogP = 2.67, < 0.1 mg/mL at pH 7), limiting its administration orally or systemically^31^. In NMP, MK-2048 had a significantly higher saturation solubility of 715 ± 5 mg/mL (Supplementary Table 1). Dolutegravir, is another second-generation integrase strand transfer inhibitor (INSTI) highly potent against HIV-1 with an in vitro protein-adjusted IC90 for HIV-1 of 0.064 μg/mL. Dolutegravir (DTG) had a saturation solubility in NMP of 255 ± 4 mg/mL (Supplementary Table 1). Rilpivirine (RPV), had a saturation solubility in NMP of 228 ± 5 mg/mL. Darunavir, Ritonavir, and Atazanavir are hydrophobic drugs like MK 2048, DTG and RPV. These three drugs also exhibited very high solubility in NMP ranging from 328 ± 2 mg/mL (atazanavir) to 511 ± 2 mg/mL (darunavir). Raltegravir, a hydrophilic drug, exhibited lower solubility in NMP (54 ± 2 mg/mL) compared to MK-2048 and DTG. Lamivudine and Stavudine are also hydrophilic drugs exhibited high solubility in NMP (250 ± 3 mg/mL and 248 ± 3 mg/mL, respectively). The average solubility of the antiretroviral drugs tested in NMP was ~260 mg/mL (Supplementary Table 1). Collectively, these data showed that a wide range of ARVs from multiple classes exhibited high solubility in NMP making it an ideal solvent for ISFI formulations for long-acting delivery of ARVs. MK-2048 and DTG were initially selected to test in the ultra-long-acting injectable formulation.

### Density and rheology of ISFI formulations

To measure density and viscosity of ISFI formulations seven different ISFI formulations were prepared by dissolving PLGA (50:50 LA/GA, 27 kDa) in NMP at a range of weight ratios. Placebo formulations containing 1:2, 1:4, 1:9, 1:16, and 1:30 weight ratios (w/w) of PLGA to NMP and ISFI formulation containing MK-2048 (0.9:1:2 w/w/w ratio of MK-2048/PLGA/NMP) and DTG (0.3:1:2 w/w/w ratio of DTG/PLGA/NMP) were evaluated. The density of placebo formulations decreased slightly with increasing concentration of NMP in the formulation. Addition of drug to the formulation had no or minimal effect on formulation density (Table 1). The viscosity of the placebo formulations increased with increasing amount of PLGA and ranged from 2.6 cP for 1:30 w/w PLGA/NMP to 475 cP for 1:2 w/w PLGA/NMP (Table 1). ISFI formulations (1:2 w/w PLGA/NMP) with MK-2048 (300 mg/mL) and DTG (100 mg/mL) had viscosity of 1242 cP and 845 cP, respectively. Compared to placebo formulation with the same PLGA/NMP ratio the viscosity increased 2.6-fold and 1.8-fold for MK-2048 ISFI and DTG ISFI, respectively. Despite the increase in viscosity, both drug ISFIs were syringeable and could be readily administered subcutaneously to mice.

**Table 1:** Density and rheology of ISFI formulations

ISFI Formulation (1 mL)	Density^a^ (g/mL)	Temp (°C)	Spindle speed (g^c^)	Shear rate (sec^−1^)	Viscosity^b^ (cP)	Torque (%)	Shear stress (Pa)
1:2 w/w PLGA/NMP	1.106	25	2.7E−04	3.8	475	79	18.2
(placebo)		37	2.7E−04	3.8	345	58	13.3
1:4 w/w PLGA/NMP	1.047	25	6.7E−03	19.2	68.1	56.6	13.1
(placebo)		37	2.7E−02	38.4	46.3	77.1	17.8
1:8 w/w PLGA/NMP	1.038	25	2.7E−02	38.4	15.4	25.7	5.92
(placebo)		37	2.7E−02	38.4	10.5	17.5	4.03
1:16 w/w PLGA/NMP	1.015	25	2.7E−02	38.4	5.3	8.8	2.0
(placebo)		37	2.7E−02	38.4	3.7	6.1	1.4
1:30 w/w PLGA/NMP	1.002	25	2.7E−02	38.4	2.6	4.3	1.0
(placebo)		37	2.7E−02	38.4	1.7	2.9	0.7
0.9:1:2 w/w/w MK-2048/PLGA/NMP MK-2048 ISFI	1.147	25	4.3E−05	1.5	1242	82.7	19.1
		37	4.3E−05	1.5	584.9	39.0	9.0
		37	2.7E−04	3.8	590.3	98.4	22.7
0.3:1:2 w/w/w	1.113	25	6.7E−05	1.9	845	70.5	16.2
DTG/PLGA/NMP		37	6.7E−05	1.9	445	37	8.5
DTG ISFI		37	2.7E−04	3.8	444	74	17

^a^Density measurements were done in triplicates (*n* = 3, standard deviation (s.d.) < 0.01)

^b^Viscosity measurements were done in triplicates (*n* = 3 readings/sample, s.d. < 0.01)

^c^Spindle radius 24 mm

### Stability studies

To determine the shelf-life of MK-2048 and DTG ISFIs, stability studies were carried out at three different storage conditions (5 °C ± 2 °C, 25 °C ± 2 °C, 40 °C ± 2 °C/75% relative humidity (RH)). Stability was determined for both the ISFI formulation (change in physical appearance) and drug formulated in the ISFI (drug concentration, physical/chemical integrity, activity). ISFI formulations were prepared with MK-2048 (300 mg/mL, 0.9:1:2 w/w/w of MK-2048/PLGA/NMP ratio, PLGA 27 kDa) and DTG (100 mg/mL, 0.3:1:2 w/w/w of DTG/PLGA/NMP ratio, PLGA 27 kDa) and sample aliquots were collected longitudinally over four months to quantify drug concentration and presence of any drug degradation peaks by high-performance liquid chromatography (HPLC). Table 2 (Supplementary information) shows the residual drug at each time point relative to initial drug concentration (time 0 h). For MK-2048 ISFI, the residual drug was 98.5% at day 30 for the formulation stored at 5 °C and to 97.2% and 95.7% after 4 months for formulations stored at 25 °C and 40 °C/75% RH, respectively. For DTG ISFI, the residual drug decreased to 97.8% at day 30 for the formulation stored at 5 °C and to 95.7 and 91.4% after 4 months for formulations stored at 25 °C and 40 °C/75% RH, respectively. ISFI solutions were also monitored for changes in appearance, color, or consistency. No change in physical appearance or consistency was observed after 0, 1, 3, or 4 months of storage at 25 °C or 40 °C/75% RH storage conditions. However, for the formulation stored at 5 °C, the solution turned turbid by day 30 with both MK-2048 and DTG likely due to drug precipitation from the solution or phase separation between PLGA and NMP. These results indicate that both MK-2048 and DTG formulations maintain their stability if stored at room temperature conditions (25 °C) for at least 4 months. The slight decrease in drug concentration (<4% for MK-2048 at 40 °C/75% RH and <7% for DTG at 40 °C/75% RH) over four months was likely due to drug degradation at lower pH as a result of PLGA hydrolysis under the stability study conditions (Supplementary Table 2). However, no degradation peaks were detected by HPLC analysis for MK-2048 and DTG (Supplementary Fig. 1).

### Physical stability of MK-2048 and DTG in ISFI formulation

**Fig. 1:**
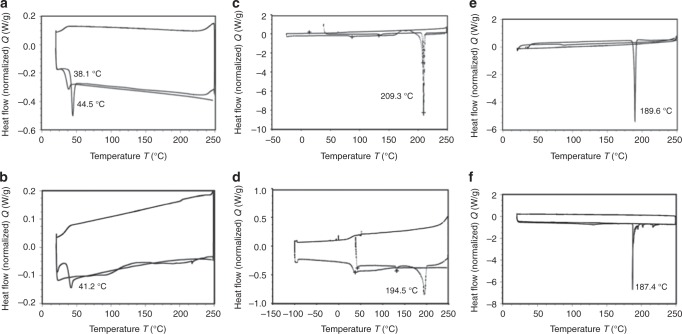
Drug stability in ISFI formulation by DSC analysis. DSC thermograms: **a** PLGA (neat; 50:50 LA:GA, MW 27 kDa), **b** 1:2 PLGA/NMP Placebo ISFI, **c** MK-2048 (neat), **d** 0.9:1:2 MK-2048/PLGA/NMP ISFI, **e** DTG (neat), **f** 0.3:1:2 DTG/PLGA/NMP; the PLGA peak at ~40–50 °C is not visible due to scale relative to the strong DTG peak. Endodermic peaks with the respective glass transition and melting temperatures are indicated in each panel

The physical state of the drug in the implant, i.e., crystalline state or molecularly dispersed can influence the extent of burst release and release rate of a drug from the formed implant^34^. We used differential scanning calorimetry (DSC), a thermoanalytical technique to evaluate the physical state and melting temperature of PLGA and drug in solidified ISFIs. Figure 1a–f shows DSC thermograms of individual neat (reagent only) excipients (PLGA, MK-2048, and DTG), and placebo implant or drug implants prepared by solidification of liquid formulation (20 µl) upon injection to phosphate-buffered saline (PBS). Thermograms of neat PLGA and placebo implant (1:2 w/w PLGA/NMP) showed a broad endothermic peak at 40–50 °C for PLGA and 41–44 °C for placebo implant (Fig. 1a, b) reflecting the amorphous nature of the PLGA 50:50 (LA:GA)^34^. Neat MK-2048 and DTG gave sharp endothermic peaks at the glass transition temperature 209.3 °C and 189.6 °C, respectively (Fig. 1c, e). MK-2048 and DTG in ISFI formulations (0.9:1:2 and 0.3:1:2 w/w/w drug/PLGA/NMP ratios) showed endothermic peaks for MK-2048 and DTG at 194.5 °C and 187.4 °C, respectively (Fig. 1d, f). This demonstrated that no chemical interaction took place between the drug and polymer indicating that MK-2048 and DTG are both stable in the ISFI formulation. Figure 1d also showed a PLGA endotherm at 42.4 °C which showed that the glass transition temperature of PLGA is maintained in the presence of MK-2048 indicating the stability of PLGA in the presence of drug. Interestingly, we found that the melting peak intensity for PLGA (Fig. 1a, b) and MK-2048 (Fig. 1c, d), but not DTG (Fig. 1e, f), was substantially smaller in the ISFI formulations compared to neat PLGA and neat MK-2048. This suggests that PLGA and MK-2048 were less crystalline and more amorphous when formulated in the ISFI compared to DTG.

### Drug activity after long-term storage in ISFI formulation

DTG-ISFI formulation was stored for 6 months at 25 °C and then solidified in PBS. The concentration of DTG eluted from the solidified implant after 24 h incubation in PBS was determined by HPLC and its HIV inhibitory activity was assessed using TZM-bl cells as described in the methods section. The effective concentration of DTG eluted from the implant required to inhibit virus replication by 50% (IC50) was 2.1 ng/ml (Supplementary Fig. 2). This value is consistent with the IC50 previously reported for DTG^35,36^ and it was not significantly different from the activity of a freshly prepared solution of DTG (Supplementary Fig. 2).

### Scanning electron microscopy (SEM) analysis

**Fig. 2:**
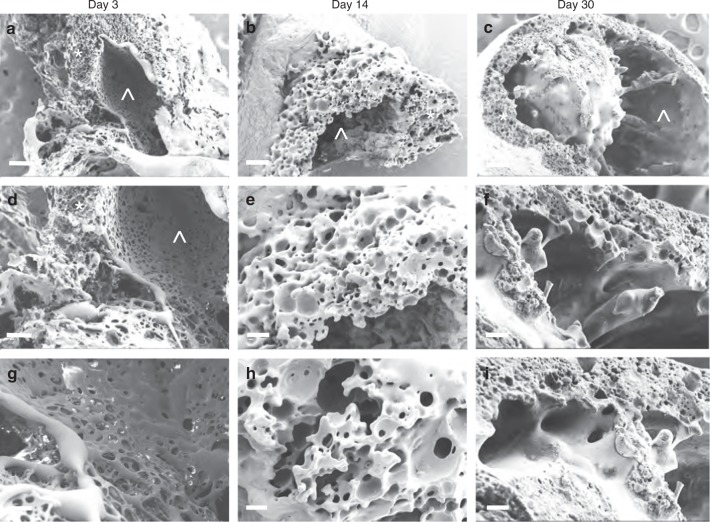
Effect of PLGA degradation on implant microstructure. SEM cross-section images of placebo-ISFIs (1:2 w/w PLGA/NMP, PLGA MW 27 kDa) over a 30 day period (*n* = 3). **a**–**c** low magnification image (×100 ) of the entire implant (scale bar = 100 µm), **d**–**f** higher magnification (×200) of the implant shell (shell thickness was measured using SEM scale; scale bar = 50 µm), **g**–**i** higher magnification (×500) of the center of the implant (scale bar = 20 µm). **a**, **d**, **g** Implants imaged at day 3 post incubation in 0.01 M PBS pH 7.4 at 37 °C; **b**, **e**, **h** implants imaged at day 14 post incubation; **c**, **f**, **i** implants imaged at day 30 post incubation. Representative image for each magnification and time point is shown (*n* = 3). Symbols represent implant shell (*) and pore (^)

The release kinetics of drugs from ISFIs is largely influenced by the microstructure of the implant formed in vitro and in vivo. The microstructures of implants can be influenced by factors including the polymer type, solvent and drug properties, miscibility of the polymer with the solvent, the rate of phase inversion, the effect of the injection site, and the rate of implant degradation^24,26–28,37–41^. Scanning electron microscopy (SEM) imaging was used to investigate the effect of PLGA erosion on the microstructure environment of the implant. Samples were prepared by injecting 20 µL of ISFI formulation (1:2 w/w PLGA/NMP, PLGA MW 27 kDa, 1 mL) into PBS and incubating for 3, 14, and 30 days at 37 °C (*n* = 3 per time point). Upon injection of the ISFI formulation into PBS, the NMP diffused into the aqueous medium and the ISFI solidified. Cryosectioning of the implant formed in vitro was not possible until day 3 due to implant distortion during the freeze-drying process part of the sample preparation for SEM imaging^41,42^. As shown in Fig. 2, at day 3, the implant consisted of a central pore caused by the residual NMP. This central pore is surrounded by a thick polymer outer shell of highly interconnected porous structures consistent with an instantaneous precipitation of PLGA from highly miscible solvent like NMP^41,42^. Erosion of implant was observed by decreasing thickness of the outer shell of the implant from 500–600 μm at day 3 to 200–300 μm at day 30. Additionally, changes in the porous structure of the implant shell were noticed. The diameter of the pores in the shell was in the range of 10–40 μm at day 3. At day 30, large pores were observed with a diameter in the range of 40–100 μm and length of 40–400 μm, respectively. The microstructure of implants at each time point did not differ between samples (*n* = 3).

**Fig. 3:**
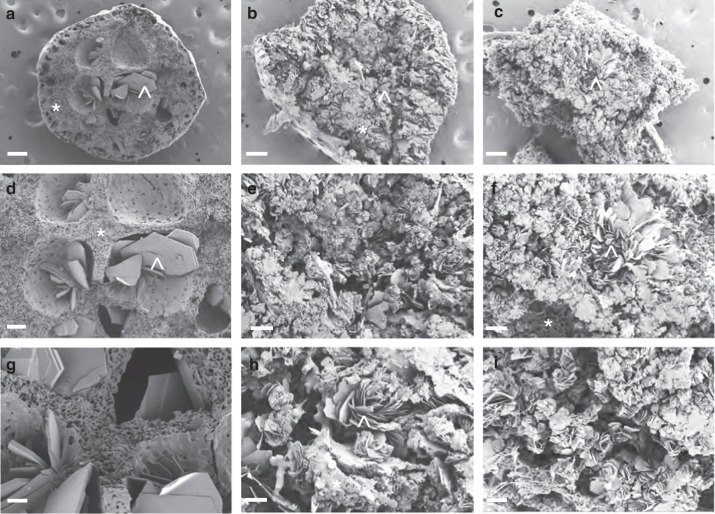
Effect of PLGA/NMP ratio on the microstructure of the DTG implants. SEM cross-section images of DTG-ISFIs after drug release for 30 days (*n* = 3). Each column is a representation of ISFIs containing a different ratio of PLGA to NMP. **a**, **d**, **g** 1:2 w/w PLGA/NMP ratio, **b**, **e**, **h** 1:4 w/w PLGA/NMP ratio, and **c**, **f**, **i** 1:8 w/w PLGA/NMP ratio. Row 1 (**a**, **b**, **c**) is a low magnification image (×100, scale bar = 100 µm) of the entire implant, Rows 2 **(d**, **e**, **f**) and 3 (panels **g**, **h**, **i**) are higher magnification images (×200 , and ×500 , respectively, scale bar = 50 µm, 20 µm) focusing on the center of the implant. **a** 1:2 PLGA/NMP ×100 , **b** 1:4 PLGA/NMP ×100 , **c** 1:8 PLGA/NMP ×100  (**a**–**c** scale bar = 100 µm), **d** 1:2 PLGA/NMP ×200, **e** 1:4 PLGA/NMP ×200 , **f** 1:8 PLGA/NMP ×200 (**d**–**f** scale bar = 50 µm), **g** 1:2 PLGA/NMP ×500 , **h** 1:4 PLGA/NMP ×500 , **i** 1:8 PLGA/NMP ×500 (**g**–**i** scale bar = 20 µm). Representative image of each magnification and PLGA/NMP ratio is shown. Symbols represent PLGA (*) and DTG (^)

The effect of PLGA to NMP ratio on the microstructure of implants was also investigated by SEM imaging. DTG was used as a model drug for DTG-ISFI formulations (100 mg/mL DTG) containing 1:2, 1:4, and 1:8 weight ratios of PLGA to NMP. Solidified implants were incubated for 30 days in PBS at 37 °C and the cross-sectional images of implants were collected. DTG containing implants in Fig. 3a, d, g can be directly compared to the placebo implant of same PLGA/NMP ratio in Fig. 2c, f, i (1:2 w/w PLGA/NMP). Interestingly, the presence of DTG resulted in lower erosion of outer shell and a smaller central pore compared to the placebo implant. DTG formed rose-like shapes within the microstructures of the implant (compare Figs. 2 and 3), likely due to the crystalline nature of DTG in the implant demonstrated by the DSC analysis. Increased amounts of NMP in the formulations with PLGA/NMP 1:4 and 1:8 w/w resulted in smaller and high density rose-like structures that were distributed throughout the implant (compare Fig. 3a, d, g with 3b, e, h and 3 c, f, i). The presence of more NMP also led to greater drug dissolution and mixing within the PLGA matrix, which resulted in smaller microstructures within the implant when the NMP diffused out of the solution and the PLGA/DTG phase-inverted and formed a solid implant. As shown in Fig. 3a–c, the 1:2 w/w PLGA/NMP DTG-ISFI had a thicker outer shell structure at day 30 compared to the 1:4 and 1:8 DTG-ISFI formulations (Fig. 3b, c), suggesting greater erosion of PLGA in implants originated from ISFI formulation with higher content of NMP.

### In vitro release studies

**Fig. 4:**
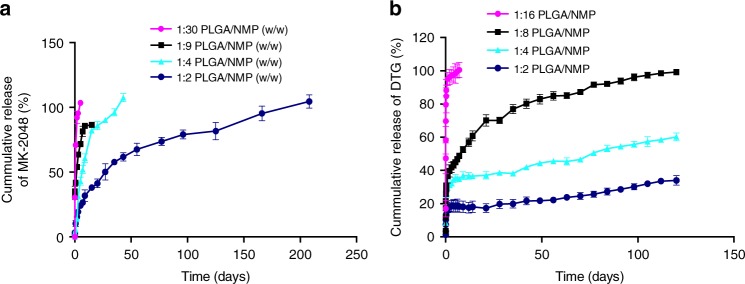
In vitro release kinetics. Cumulative release of MK-2048 over the course of 220 days (**a**) and DTG over the course of 120 days (**b**) from ISFI formulations containing varying ratios of PLGA to NMP. Drug release studies were carried out under sink conditions in 0.01 M PBS pH 7.4 + 2% solutol at 37 °C. Drug concentration was maintained constant across all PLGA/NMP ratios for each drug. All error bars are equivalent (s.d. positive and negative values) and represent standard deviation with *n* = 3. Release kinetics were significantly different across various PLGA/NMP ratios for both MK-2048 and DTG-ISFIs and across drugs at the same PLGA/NMP ratio (1:2 and 1:4 w/w PLGA/NMP) (Kruskal–Wallis test, *p* < 0.0001). Source data are provided as a Source Data file

ISFIs have characteristic release kinetics that include three phases: burst release, diffusion, and degradation^41,43^. The effect of PLGA to NMP weight ratio on drug release kinetics was investigated with MK-2048 or DTG-ISFIs. As shown in Fig. 4, both drugs exhibited the same relationship correlating release kinetics to the ratio of PLGA to NMP. Formulations containing a higher amount of NMP exhibited higher burst release and faster release kinetics. For both drugs, burst release occurred over the first 24 h post formulation injection into the release medium. For MK-2048-ISFIs, burst release ranged between 95 and 16% for formulations containing 1:30 w/w PLGA/NMP and 1:2 w/w PLGA/NMP respectively. For DTG-ISFIs, burst release ranged between 85 and 8% for formulations containing 1:16 w/w PLGA/NMP and 1:2 w/w PLGA/NMP respectively. When comparing the burst release of the two drugs, MK-2048 exhibited a larger burst release across all formulations compared to DTG. This could be attributed to the higher solubility of MK-2048 in NMP (715 ± 5 mg/mL) compared to DTG (255 ± 4 mg/mL) (Table 1, Supplementary Information), and to the amorphous nature of MK-2048 in the ISFI formulation as per the DSC analysis (Fig. 1d). For MK-2048, 100% release was reached by day 40 or earlier for three of the PLGA/NMP ratios investigated (1:30, 1:9 and 1:4 w/w). At the lowest ratio of PLGA/NMP (1:2 w/w), MK-2048 release reached 100% by day 208 (Fig. 4a). The release rate of MK-2048 within the zero order kinetics ranged between 17.4 μg/week (1:2 w/w PLGA/NMP) to 43.6 μg/week (1:8 w/w PLGA/NMP). For DTG, formulations containing 1:16 and 1:8 w/w PLGA/NMP reached 100% release by day 30 and day 120, respectively. At day 120, the remaining DTG-ISFIs (1:4 and 1:2 w/w PLGA/NMP) reached 60 and 34%, respectively (Fig. 4b). The release rate of DTG within the zero order kinetics ranged between 9.4 μg/week (1:2 w/w PLGA/NMP) and 33 μg/week (1:8 w/w PLGA/NMP). Overall, DTG exhibited slower release kinetics compared to MK-2048 which could be attributed to its crystalline nature in the ISFI (Fig. 1f) and its lower solubility in NMP compared to MK-2048. The release kinetics of the various PLGA/NMP ratios for both MK-2048-ISFI and DTG-ISFIs (in the 1:2 and 1:4 PLGA/NMP ISFIs) were statistically significantly different (Kruskal–Wallis test, *p* *<* 0.0001). For both drugs, the data showed the ability to fine-tune the release kinetics in vitro by changing the ratio of PLGA to NMP. The difference in the release kinetics of MK-2048 and DTG was attributed to factors including the difference in drug loading (MK-2048 300 mg/mL, DTG 100 mg/mL), the affinity of the drug to NMP and PLGA, the solid state of drug in the ISFI (crystalline vs. amorphous), and the dissolution of the drug from the ISFI over time. Both drugs exhibited long-term release profiles from the ISFI demonstrating the promise of using these formulations as ultra-long-acting drug delivery systems.

### In vivo ultrasound imaging

**Fig. 5:**
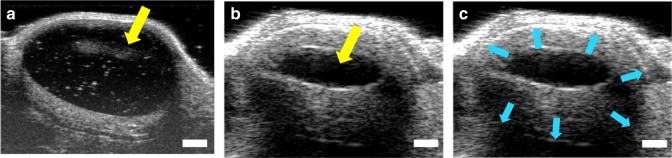
Ultrasound (US) images of the ISFI after subcutaneous injection. ISFI formulation containing 1:2 w/w PLGA/NMP was injected (65 µL) s.c. into nude (nu/nu) mice (*n* = 7) and imaged using volumetric ultrasound. Images illustrate 2D slices from one plane through the same subcutaneous implant volume at days 0 and 2 (**a**, **b**, respectively). The black center region (yellow arrow) indicates the portion of the implant remaining in the liquid state (presumably, NMP solvent), which decreased after initial injection. **c** is the same implant as **b**, except with green arrows delineating the outer implant boundary, indicated by a fine grayscale demarcation in the ultrasound image. Volume measurements were obtained by a reader who measured the approximate implant dimensions on two orthogonal image planes, resulting in length, width and depth measurements to estimate ellipsoid volume (volume of the liquid core was not separately quantified in this study). Scale bars indicate 1 mm

**Fig. 6:**
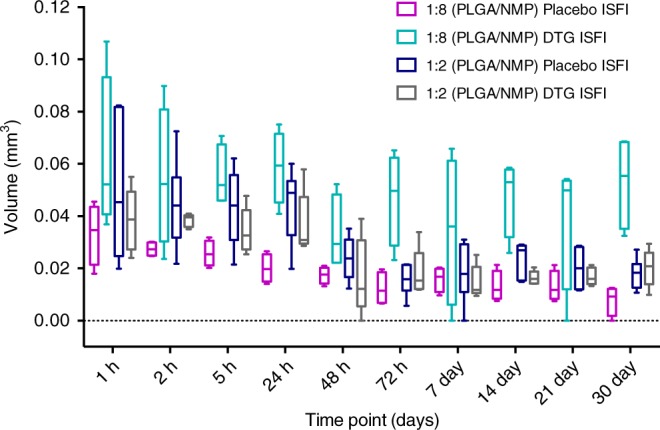
Analysis of the change in implant volume over time. Measurement of the implant volume decrease that occurs over 30 days calculated from 2D in vivo ultrasound images for four different ISFI formulations (*n* = 7 mice per group, see also Supplementary Figs. 4 and 5). Boxes represent 25th to 75th percentiles, and whiskers the range from the minimum to maximum values. Source data are provided as a Source Data file

Ultrasound (US) imaging^24,39–41^ was used to assess implant bioerosion by measuring the volume of the four different ISFI formulations (1:2 and 1:8 w/w PLGA/NMP placebo and DTG-ISFIs) in vivo over 30 days. The effect of the PLGA/NMP ratio (1:2 vs. 1:8 w/w PLGA/NMP) and the effect of the presence of drug (placebo vs. DTG ISFIs) on depot degradation in vivo were assessed by measuring volume change of implants over 30 days. In the first 3 days, the US images depicted the natural influx of fluid and hardening of the implant over time from the outside to the inside, with a corresponding increase in ‘white’ contrast caused by the PLGA polymer coming out of solution (Fig. 5a, b). Over time all four formulations exhibited a reduction in implant volume (Fig. 6). Data in Fig. 6 indicated a large variance in implant volume measurements across animals. Notably, the 1:8 PLGA/NMP placebo formulation had a significantly smaller volume than the 1:8 DTG-ISFI formulation at all time the points analyzed except for the 48 hr and 7-day time points (p < 0.05). Also, the 1:8 DTG-ISFI formulation was significantly smaller than the 1:2 DTG-ISFI formulation at the 1 h, 72 h, and 14 day time points. A plot of implant volume change over time for individual mice is included with the supplementary information (Supplementary Fig. 4). In addition, a plot of raw and model-estimated mean % volume of implant, by group and time is included in the supplementary information (Supplementary Fig. 5). When comparing the volume change at day 30 for the 1:2 PLGA/NMP ISFI formulation with and without DTG, the mean volume at day 30 (*n* = 7) relative to day 0 (100%) was 48 and 39% for placebo and DTG ISFIs respectively (Supplementary Fig. 5). This data shows that the volume decrease due to PLGA degradation was ~1.73%/day for placebo implants compared to 1.37%/day for DTG implants. This was calculated based on the 52% (V_day30_ = 48% V_day0_) and 41% (Vd_ay30_ = 39% V_day0_) volume reduction over 30 days for placebo and DTG ISFIs, respectively. These data support the hypothesis that higher solvent content leads to greater change in implant volume, and that the placebo ISFI erodes more rapidly than ISFI with drug. When an ISFI containing a higher amount of NMP is injected, a larger amount of solvent diffused out leaving behind a smaller implant of PLGA (placebo) or PLGA/drug (DTG-ISFI). In addition, when more solvent diffused out, a larger volume of water diffused into the polymer implant, which in turn accelerated the ester hydrolysis of the PLGA implant. The ability to control the rate of implant erosion by changing the ratio of PLGA/NMP demonstrates the flexibility to fine-tune the formulation.

### In vivo pharmacokinetic studies

**Fig. 7:**
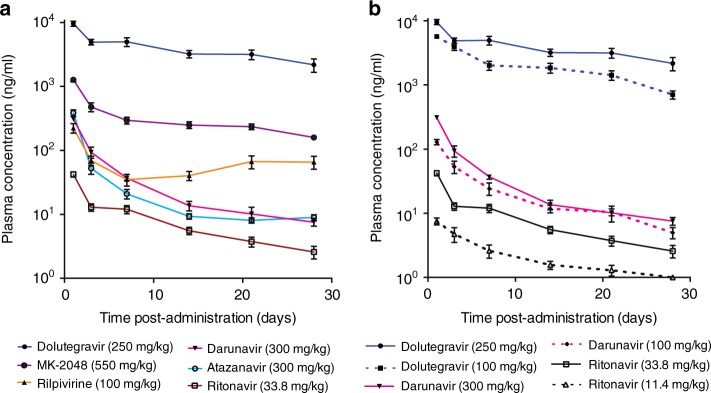
Plasma concentration of various ARVs formulated as ISFI. ISFI formulations were administered subcutaneously to NSG mice (*n* = 4) and plasma samples were collected longitudinally over 30 days. **a** Plasma concentrations of individual ARVs, **b** comparison of ISFI made using two different concentrations of dolutegravir (250 and 100 mg/kg), darunavir (300 and 100 mg/kg), and ritonavir (33.8 and 11.4 mg/kg). Mean plasma concentrations + s.e.m. are shown. Protein-adjusted IC90 (PA-IC90) values are: dolutegravir 64 ng/ml, MK-2048 33 ng/ml, rilpivirine 12 ng/ml, darunavir 2.4 ng/ml, and atazanavir 14 ng/ml. Since ritonavir is used as a booster in these formulations its IC90 is not indicated. Source data are provided as a Source Data file

**Fig. 8:**
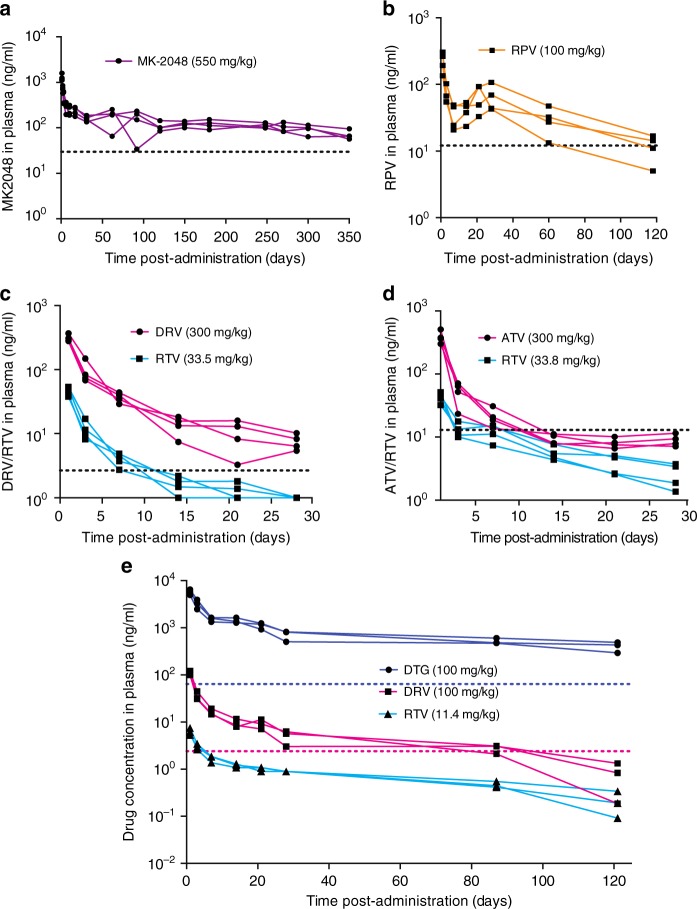
In vivo release of various ARVs formulated individually or in combination. Indicated dose of antiretrovirals formulated individually (**a**, **b**) or in combination (**c**–**e**) as ISFI was injected subcutaneously into NSG mice and plasma concentrations were analyzed longitudinally. Plasma concentrations for each individual mouse are shown. **a** MK2048, *n* = 4. **b** Rilpivirine (RPV), *n* = 4. **c** Co-formulated darunavir (DRV) and ritonavir (RTV), *n* = 4. **d** Co-formulated atazanavir (ATV) and ritonavir (RTV), *n* = 4. **e** Co-formulated dolutegravir (DTG), darunavir (DRV) and ritonavir (RTV), *n* = 3 PA-IC90 of drugs in ISFIs are indicated with dotted lines in each panel and correspond to the following values: MK-2048 33 ng/ml (**a**), rilpivirine 12 ng/ml (**b**), darunavir 2.4 ng/ml (**c**, **e**), atazanavir 14 ng/ml (**d**), dolutegravir 64 ng/ml (**e**). Since ritonavir is used as a booster in these formulations its IC90 is not indicated

To assess drug release kinetics in vivo using the ISFI formulations, initial pharmacokinetic studies were carried out using single-drug ISFIs (Figs. 7a and 8). Formulations were prepared with six individual antiretroviral drugs in 1:2 w/w PLGA/NMP (PLGA 27 kDa). For each formulation, the indicated dose of the individual drug was injected subcutaneously into NOD scid gamma chain knockout mice (NSG) mice (*n* = 4) based on their body weight, and plasma samples were collected on day 1, 3, 7, 14, 21, and 30 and analyzed for drug concentration (Figs. 7a and 8). Dolutegravir and darunavir, together with ritonavir were administered at two different doses each to assess the effect of administered dose on drug release kinetics (Fig. 7b). Initial estimates for PK parameters were obtained through non-compartmental analysis (NCA) using WinNonlin Phoenix 6.1 (Pharsight, Mountain View, CA) on the composite median PK profile. Plasma concentrations were quantified using a validated high-performance liquid chromatography-tandem mass spectrometry LC/MS-MS method^44^ and were plotted over time in Fig. 7. The median composite concentration vs time profiles suggested a multiphasic elimination for all six individual drug ISFI formulations. After an initial rapid decline in plasma concentrations, drug release approached zero order kinetics (Fig. 7). For dolutegravir, darunavir, and ritonavir, administration of higher dose led to higher plasma concentration, however, a formal assessment of dose proportionality was not conducted (Fig. 7b). Comparing dolutegravir, rilpivirine and darunavir when administered at equal dose (100 mg/kg), dolutegravir exhibited the highest plasma concentrations throughout the entire duration of the study. These results demonstrated that in vivo pharmacokinetics were drug dependent and exhibited different profiles when administered in the same ISFI formulation and equivalent dose. More hydrophobic drugs like atazanavir (LogP 4.5), rilpivirine (LogP 4.86) and ritonavir (LogP 3.9) exhibited a faster decrease in plasma concentration within the first 24–48 h (Fig. 7a). This can potentially be attributed to their high solubility in NMP and lower affinity for the PLGA matrix resulting in a greater burst release.

The ultra-long-acting release of drugs beyond 30 days and the ability to formulate multiple drugs in a single ISFI formulation are illustrated in Fig. 8. This data shows that all drugs exhibited sustained plasma concentrations when administered alone or in combination with other drugs. Plasma concentrations of MK-2048 and dolutegravir dosed at 550 mg/kg and 250 mg/kg respectively were 3× and 10× greater than the protein adjusted (PA)-IC90 respectively for at least 5 months post-administration. Both MK-2048 and dolutegravir exhibited sustained plasma concentrations for four months (DTG-ISFI) to up to one year (MK-2048-ISFI). Rilpivirine at a dose of 100 mg/kg exhibited a faster clearance with plasma concentrations dropping below the PA-IC90 by day 120 post-administration (1 of 4 mice had RPV below IC90 at day 60 post-administration). Darunavir was dosed at 300 mg/kg and exhibited an initial first order decline in plasma followed by sustained release of plasma concentrations at or above the PA-IC90 for at least 30 days. Fast clearance was also observed for atazanavir dosed at 300 mg/kg with plasma concentrations dropping below the PA-IC90 at day 10 post-administration. To establish the ability of the ISFI to deliver multiple drugs in a single injection, we combined in one formulation darunavir with ritonavir, in a second formulation we combined atazanavir with ritonavir, and in a third formulation we combined the integrase inhibitor dolutegravir with darunavir and ritonavir. Ritonavir in these formulations was included as a booster for the protease inhibitors^45^. Figure 8c, d, e show that combination of multiple ARV in single injection results in sustained release of all three drugs. These results demonstrate the ability to co-formulate multiple drugs into a single ISFI solution and to maintain high concentrations of drug in the plasma for several months.

### Termination of drug delivery by ISFI removal

**Fig. 9:**
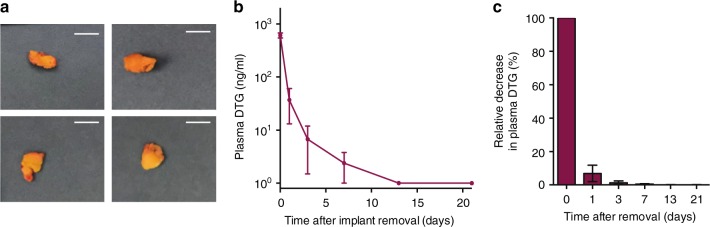
Removal of DTG-ISFI results in rapid reduction of plasma drug levels. Mice were administered DTG-ISFI subcutaneously (250 mg/kg, *n* = 5). Four months later, implants were removed and levels of DTG in plasma were monitored for 3 weeks. **a** Implants removed from mice 4 months post injection; bar indicates 5 mm. **b** plasma concentration of DTG after implant removal (*n* = 5, mean ± s.e.m.). **c** percent decrease in plasma DTG concentration relative to day 0 (*n* = 5, mean ± s.e.m.). Source data are provided as a Source Data file

To determine the efficiency and kinetics of termination of drug delivery after ISFI removal, we administered DTG-ISFI (250 mg/kg) subcutaneously to five NSG mice. Four months after ISFI administration, the median concentration of DTG in plasma was 629 ng/ml (range 529–822 ng/ml) (Fig. 9). Implants were then removed by making a small incision into the mouse skin next to the implant injection site (Fig. 9a). Plasma DTG concentration was measured at 1, 3, 7, 14, and 21 days after implant removal (Fig. 9b). Interestingly, 24 h after implant removal (first time measured), DTG plasma concentrations dropped 36-fold to a median concentration of 17.3 ng/ml (range 5.4–132.0 ng/ml). At this time point, four out of the five animals had DTG plasma levels below its PA-IC90 (64 ng/ml). By day 3, the median DTG concentration was reduced to 1.8 ng/ml (range 1–27.6 ng/ml) (Fig. 9c). By day 7, DTG plasma concentrations were below the limit of detection in all but one mouse that had a DTG concentration of 8.0 ng/ml. This mouse had DTG plasma levels below the limit of detection by day 14 (Fig. 9b). Analysis of residual DTG in the removed implants and comparison to the dose administered showed that 4 months post ISFI administration, 30% of the original amount of DTG (range 28.6–33.7%) was still available in the implants. These data show that sustained release of drug from the ISFI can be efficiently stopped by removal of the implant.

## Discussion

Developing a polymer-based biodegradable implant that can provide sustained delivery of drugs over weeks or months after a single administration is of high importance in a wide range of medical applications that require frequent drug dosing regimens. Of particular interest in this study, various antiretroviral drugs were tested as single and/or combination ISFI. An ultra-long-acting ISFI was developed with the ability to formulate a number of different antiretroviral drugs at high concentrations that can translate to possible applications in humans. This ternary system comprises a biodegradable polymer (PLGA), a biocompatible and water-miscible solvent (NMP), and a drug in a homogeneous solution that becomes a solid implant in situ when administered subcutaneously. Drug stability assessed by DSC analysis shows that drug endotherms are maintained when formulated in the ISFI. The physical and chemical properties of drug ISFIs are stable over 4 months when stored at room temperature. Moreover, drug (DTG) activity based on its ability to inhibit HIV infection of TZM-bl cells is maintained post storage of the DTG-ISFI formulation at 25 °C for 6 months demonstrating the long-term stability of drug in the ISFI formulation. In vitro release studies for MK-2048 or dolutegravir ISFIs demonstrate the ability to fine-tune the release kinetics by changing the ratio of PLGA to NMP. Moreover, when formulated in a 1:2 w/w PLGA/NMP, MK-2048, and DTG exhibit sustained release for up to one year and 4 months respectively with zero order release kinetics. Sustained drug delivery for weeks and months after a single injection of a long-acting drug formulation is desirable for many clinical applications. However, long-term sustained drug delivery can be detrimental especially in cases where treatment needs to be terminated (e.g., pregnancy, negative response to drug, change of therapy regimen). In addition, long-acting formulations can deliver unwanted subtherapeutic drug concentrations for long periods of time often exceeding the treatment period. Therefore, the ability to remove the implant and efficiently terminate drug delivery is of great importance. Our results show that ISFIs can be readily removed and that their removal results in effective termination of drug delivery.

Ultrasound imaging was used to non-invasively follow the formation and bioerosion of the implant in vivo. Data from in vivo ultrasound imaging show that the rate of volume change can be tuned by changing formulation parameters like the ratio of PLGA to NMP. ISFI formulations containing a higher amount of NMP (PLGA/NMP 1:8 v/v) exhibited greater volume decrease at day 30 (70%) compared to formulations containing less NMP (PLGA/NMP 1:2 w/w) with only 52% volume decrease by day 30. In principle, ultrasound imaging could be used to localize the implant and surgically remove it in the case of pregnancy, adverse or allergic reaction or other need to terminate the treatment. In vivo pharmacokinetic studies showed plasma concentrations remained 1–2 logs above the PA-IC90 for MK-2048 and dolutegravir for over eleven and four months respectively. Of the six drugs individually tested in vivo, dolutegravir exhibited the highest plasma concentrations. The in vivo pharmacokinetic studies also showed the ability to co-deliver two or three antiretroviral drugs in a single ISFI and achieve sustained plasma concentrations for the individual drugs. All ISFI formulations were well-tolerated in vivo with no signs of local inflammation or change in body weight. Collectively, these data and recently published data^46,47^ demonstrate the flexibility of the ISFI formulation to achieve target release kinetics and drug concentrations that can be translated to humans. To our knowledge, this is the first report on a polymer-based biodegradable, highly tunable, and removable implant that can be administered as a stable solution containing one or multiple antiretroviral drugs, and that can provide sustained drug delivery in vivo.

## Methods

### High-performance liquid chromatography (HPLC)

We used a well-characterized methodology for the analysis and quantitation of DTG and MK-2048^47–49^. For all other drugs a quantitative reverse-phase HPLC method was developed and validated to determine drug release in vitro from the individual ISFI formulations. The HPLC analysis was carried out with a Finnigan Surveyor HPLC system (Thermo Finnigan, San Jose, California, USA) with a Photodiode Array (PDA) Plus Detector, auto-sampler, and LC Pump Plus. The stationary phase utilized for the analysis was a Inertsil ODS-3 column (5 μm, 4.6 Å~ 150 mm, [GL Sciences, Torrance, CA]) maintained at 40 °C. Chromatographic separation was achieved by gradient elution using a mobile phase consisting of 0.1% trifluoroacetic acid in water and acetonitrile (ACN) (H_2_O/ACN 95:5 v/v). The flow rate was 1.0 mL/min and the total run time was 25 min for each 25 μL injection.

### Saturation concentration of drugs in NMP

The saturation concentration of a panel of fourteen (14) antiretroviral drugs in NMP was determined. For each drug, 100 mg was weighed into individual vials and 100 mg of NMP was added to each vial. The mixture was stirred at 40 °C for 24 h. Samples were subsequently centrifuged for 1 h at 16,000 g (Eppendorf Centrifuge 5417C, USA) to remove excess undissolved drug. Sample aliquots (1–2 mg, *n* = 4) were collected from the saturated supernatant and diluted with acetonitrile (ACN). Drug concentration in the saturated aliquots was determined by HPLC analysis.

### Preparation of ISFI formulations

In a 7 mL scintillation vial back-filled with argon, 50:50 Poly(DL-lactide-*co*-glycolide) (PLGA) was mixed with *N*-methyl-2-pyrrolidone (NMP) at various ratios of PLGA/NMP weight ratio (w/w) and allowed to dissolve by continuous mixing at room temperature to form a homogenous placebo formulation. To assess the effect of PLGA/NMP ratio on drug release kinetics, placebo formulations containing the following w/w ratios of PLGA/NMP were prepared: 1:2, 1:4, 1:8, 1:9, 1:16, and 1:30. Drug was subsequently added to the PLGA/NMP solution and stirred at 37 °C overnight to dissolve drug and produce an ISFI formulation with optimized drug concentration. To ensure complete drug dissolution in the placebo formulation and homogeneity of the drug solution, sample aliquots (1–2 mg, *n* = 4) were collected from four different areas in the drug formulation and dissolved in acetonitrile and drug concentration was quantified by HPLC analysis. A formulation was designated as homogenous when the standard deviation associated with the average concentration in all 4 aliquots was ≤5%.

Based on the saturation solubility in NMP and potency against HIV, we have selected two main drugs, MK-2048 and Dolutegravir (DTG) to carry out full in vitro characterization studies described in the following sections. ISFI formulations of MK-2048 and DTG were prepared using 1:2 w/w PLGA/NMP (PLGA MW 27 kDa) placebo formulation. The final concentrations of MK-2048 and DTG in the ISFI formulations were 300 mg/mL (30 wt% MK-2048) and 100 mg/mL (10 wt% DTG) respectively. In vitro, the percent drug entrapment of DTG in the 1:2 PLGA/NMP formulation after injection into PBS was quantified at ~98% by HPLC. Based on a 98% entrapment, DTG concentration in the solid implant when administered in vivo is estimated to be ~19 mg per 65 µL injection. To investigate the ability to formulate a wider range of antiretroviral drugs as single or multiple-drug ISFI formulations, we have investigated an additional four antiretroviral drugs (Rilpivirine, Darunavir, Atazanavir, and Ritonavir) in mouse PK studies described below.

### Density measurement and rheology analysis

The density of placebo and drug ISFIs was measured by weighing 1 mL of formulation solution in a 1 mL volumetric flask. The dynamic viscosity of various ISFI formulations was measured using a Brookfield Cone and Plate Digital Rheometer (Model: LVDV-III + CP, Middleboro MA, USA) and the reading was recorded at 25 °C and 37 °C and a spindle speed in the range of 2.7E−06–2.7E−04 g. The shear rate was varied from 0.384/s to 38.4/s for 5–60 min. The temperature was maintained at 25 and 37 °C using a circulating water bath (Brookfield TC-502, USA) surrounding the outer cylinder.

### Accelerated stability study

Formulations of MK-2048 (300 mg/mL) and dolutegravir (100 mg/mL) were prepared with varying ratios of PLGA to NMP in individual 7 mL scintillation vials back-filled with argon. The vials were stored in a desiccator and placed either at room temperature (RT) or 40 °C/75% relative humidity (RH) in a Fisher Scientific Isotemp Incubator (Pittsburgh, PA). At various intervals (0, 7, 14, 30, 90, and 120 days), samples were collected and analyzed by HPLC for drug content. Zero time samples were used as controls. The formulations were also visually inspected for any change in their physical state, i.e. color, turbidity, and consistency.

### Differential scanning calorimetry (DSC)

The DSC analyses for neat PLGA, pure drug (DTG and MK-2048), optimized placebo ISFI, and optimized drug loaded ISFIs (DTG and MK-2048 ISFIs) were carried out using a differential scanning calorimeter (TA Q200, USA). Neat PLGA and pure drugs were used as received and ISFIs were prepared by injecting a solution of the formulation (20 μL) into PBS (pH 7.4, 37 °C) and incubating for 24 h. The implant samples were collected and air-dried for 72 h prior to DSC analysis. Samples varying in weight from 1–10 mg were weighed, hermetically sealed in an aluminum pan, and placed in the differential scanning calorimeter. The samples were subsequently heated from 25–250 °C, at a heating rate of 10 °C/min, under nitrogen atmosphere (flow rate 20 mL/min). The thermograms were used to determine the peak glass transition temperature (T_g_) for PLGA and peak melting temperature (T_m_) for MK-2048 and DTG.

### Preparation of HIV-1_JR-CSF_ stocks for infection

Viral stocks of HIV-1_JR-CSF_ were generated by transfecting HIV-1_JR-CSF_ DNA^50–54^ into 293T cells (American Tissue Culture Collection, catalog number CRL-3216, passage <10) using Lipofectamine 2000 (Invitrogen). 293T cells were cultured at 37 °C, 10% CO_2_ in Dulbecco’s Modified Eagle Medium (Sigma) supplemented with 10% fetal bovine serum, 25 mM HEPES, 500 units/ml penicillin, 50 µg/ml streptomycin and 2 mM L-glutamine (Cellgro), cells were regularly checked for morphology by microscopy. Viral particles were collected in tissue culture medium that was centrifugated at 2000×*g* for 20 min at 4 °C to remove cell debris. Tissue culture infectious units (TCID)/ml of HIV were determined by titration using TZM-bl cells (NIH AIDS Research and Reference Reagent Program, catalog number 8129, passage number 2–6). Infected TZM-bl cells were stained using a solution containing 4 µM potassium ferrocyanide, 4 µM potassium ferricyanide, 2 µM magnesium chloride, 0.4 mg/ml X-gal^55,56^ and stained cells counted. TZM-bl cells were cultured at 37 °C 10% CO_2_ in TZM-bl medium (Dulbecco’s Modified Eagle Medium (Sigma) supplemented with 10% heat-inactivated fetal bovine serum, 25 mM HEPES, 500 units/ml penicillin, 50 µg/ml streptomycin and 2 mM L-glutamine (Cellgro)), and regularly checked for morphology by microscope.

### In vitro HIV-1 inhibition

TZM-bl cells (NIH AIDS Research and Reference Reagent Program, catalog number 8129, passage number 2–6), were maintained at 37 °C 10% CO_2_ in TZM-bl medium. Cells were regularly checked for morphology by microscopy. TZM-bl cells were plated in 96-well plates (1 × 10^5^ cells per well) in TZM-bl medium. The next day, the medium was removed and 100 μl of 1:100 dilutions of dolutegravir were added. Cells were incubated with DTG for 30 min and infected with 3 × 10^3^ TCIU of HIV-1_JR-CSF_ in 100 μl of TZM-bl medium containing 40 µg/ml DEAE-dextran. Approximately 48 h later, the medium was removed and the luciferase substrate One-Glo reagent (Promega, Madison, WI) supplemented with 0.2%Triton X-100 was added to allow for the measurement of luciferase activity and to inactivate virus. Luciferase activity was measured with a SpectraMax M3 Spectrometer (Molecular Devices, Sunnyvale, CA) and the results normalized to the luciferase activity of cells infected with HIV in the absence of DTG and expressed as a percentage of decrease in luciferase activity. Activity was measured in 3 independent experiments, each dilution measured in quadruplicate. The effective drug concentrations required to inhibit virus replication by 50% was calculated by fitting the data to a sigmoid dose-response curve with a variable slope using GraphPad Prism (GraphPad Software).

### In vitro cumulative drug release

To investigate the ability to fine-tune drug release kinetics with the ultra-long-acting formulation, in vitro release studies were carried out with formulations containing MK-2048 or DTG. Drug release kinetics from various ISFI formulations were evaluated by injecting the polymer solution 20 μL (20 ± 3 mg, n = 3) into 200 mL of release medium (0.01 M PBS pH 7.4 with 2% solutol HS and 0.01% NaN_3_) and incubating under sink conditions at 37 °C. Sink conditions were defined as the drug concentration at or below 1/5 of maximum solubility (i.e., ≤0.04 mg/mL MK-2048; ≤ 0.12 mg/mL DTG) in PBS/solutol at 37 °C. The saturation solubility of MK-2048 and DTG in PBS + 2% solutol at 37 °C was quantified by HPLC analysis at 174.7 ± 3.5 µg/mL and 564 ± 3.6 µg/mL, respectively. These solubilities are ~9-fold (for MK 2048) and ~6-fold (for DTG) greater than their solubility in PBS at 37 °C with no solutol (MK 2048 19 µg/mL; DTG 91 µg/mL quantified by HPLC analysis). Sample aliquots (1 mL) were collected at predetermined time points and replaced with fresh release medium (1 mL). To maintain sink conditions, the release medium was completely removed and replaced with fresh (200 mL) release medium every week. The drug concentration in the release samples was quantified by HPLC using the method described above. Cumulative drug release was calculated from the HPLC analysis and normalized by the total mass of drug in the implant. All experiments were performed in triplicate.

### Scanning electron microscopy imaging and analysis

Implant surface and microstructures were evaluated by scanning electron microscopy. First, implants were prepared by injecting 20 μL of polymer solution into 10 mL of 0.1 M PBS, pH 7.4 at 37 °C. For consistency, these studies were done using the same batch of ISFI formulation (1:2 w/w PLGA/NMP, PLGA MW 27 kDa, 1 mL). At predetermined time points (1, 3, 7, 14, and 30 days post injection), the implants were removed from the bath solution and flash-frozen then fractured over dry ice. Following freeze-fracture, implants were lyophilized for 24 h (SP VirTis Advantage XL-70, Warminster, PA). The lyophilized samples were subsequently mounted on an aluminum stub using carbon tape, and sputter coated with 5 nm of gold-palladium alloy (60:40) (Hummer X Sputter Coater, Anatech USA, Union City, CA). The coated samples were then imaged using a Zeiss Supra 25 field emission scanning electron microscope with an acceleration voltage of 5 kV, 30 µm aperture, and average working distance of 10 mm (Carl Zeiss Microscopy, LLC, Thornwood, NY).

### In vivo ultrasound imaging

A 30-day ultrasound (US) imaging study was carried out to assess the in vivo implant formation and change in volume of four ISFI formulations (placebo and DTG ISFIs with PLGA/NMP 1:2 and 1:8 w/w). In this study, sixty-five (65) µL of each formulation was injected s.c. into female nude (nu/nu) mice (*n* = 7 per group) and mice were imaged using a MS-550D Linear Array Transducer (FujiFilm VisualSonics; Toronto, Canada) on the Vevo® 2100 System (FujiFilm VisualSonics; Toronto, Canada). Imaging was performed in B-mode with a transmit frequency of 40 MHz at 10 time points: 1 h, 3 h, 5 h, 1 d, 2 d, 3 d, 7 d, 14 d, 21 d, and 3 0d to assess the effects of PLGA/NMP ratio (1:2 vs 1:8 w/w) and drug (placebo vs. DTG ISFIs) on the rate of implant volume change over 30 days. To insure a robust and unbiased approach, the four formulations prepared in the laboratory were blinded to the UNC animal core staff who performed the s.c. injections and to scientists that completed the ultrasound imaging. 3-D ultrasound scans from a pilot study demonstrated that the implants were approximately ellipsoid (Supplementary Fig. 3), and thus further study measurements were performed with two orthogonal 2-D images to visualize the subcutaneous implant. After image acquisition, the sonographer selected a region of interest on the spatially calibrated ultrasound image which approximated the boundary of the implant in the tissue. The ellipsoid value was established from the height, width, and length of the region of interest measurements. The region of interest selection was performed with 5 technical replicates for a subset of 100 of the 280 total data points. From these data, the average variance in the measured volume due to variance in the region of interest selection by the sonographer was 9.7%.

### Statistical analysis

To analyze differences in in vivo implant volumes between groups based on imaging data, a repeated-measures two-way ANOVA test was performed with respect to time point and implant formulation. A Tukey’s multiple comparisons test was then performed to assess differences in average implant volume between groups at each time point. Statistical analyses were performed in GraphPad Prism 7 (GraphPad Software, Inc., La Jolla, CA, USA).

### In vivo pharmacokinetic studies

Female NOD scid gamma (NSG) mice, 6–8 weeks (Jackson Laboratory), were housed in a pathogen-free room. All experiments involving mice were carried out with an approved protocol by the University of North Carolina Animal Care and Use Committee. In vivo pharmacokinetic (PK) studies were carried out with 3 single drug ISFI formulations and 3 combination drug ISFI formulations. Liquid ISFI drug formulations were administered subcutaneously with a 19G needle on the shaved back of anesthetized NSG mice (Supplementary Fig. 6). Peripheral blood was collected from mice into capillary tubes coated with EDTA to isolate plasma. All samples were stored at −80 °C until analysis.

### Analytical methods

Drug concentrations were measured in plasma using LC-MS/MS. Briefly, plasma samples were extracted by protein precipitation with methanol. Following precipitation, samples were mixed 1:1 with water prior to LC-MS/MS analysis. Analytes were separated on an Atlantis T3 (50 × 21.mm, 3μm) analytical column (Waters, Milford, MA, USA) prior to detection on an API-5000 triple quadrupole mass spectrometer (AB SCIEX, Foster City, CA, USA). Calibration standards and quality control samples were within 20% of nominal values with a dynamic range of 1–10,000 ng/ml. Composite concentration-time profiles were constructed for each analyte and visually inspected to make qualitative between analyte comparisons.

### Reporting summary

Further information on research design is available in the Nature Research Reporting Summary linked to this article.

## Supplementary information

Supplementary Information

Reporting Summary

Source Data File

## Data Availability

The data underlying Figs. 4, 6, 7a, 7b, 9b and 9c as well as Supplementary Fig. 2 are available in the associated source data file. All other data supporting the findings of this manuscript are available from the corresponding authors (S.R.B and J.V.G) upon reasonable request.
