# Supplementary material for: Ultra-long-acting tunable biodegradable and removable controlled release implants for drug delivery
Source: Nat Commun. 2019 Sep 20;10:4324. doi: 10.1038/s41467-019-12141-5 (PMC6754500; doi:10.1038/s41467-019-12141-5)
Supplement: Supplementary file 1 — Supplementary Information [file 41467_2019_12141_MOESM1_ESM.pdf]

Supplementary Information

Ultra-Long-Acting Tunable, Biodegradable, and Removable Controlled Release Implants for Drug Delivery.

S. Rahima Benhabbour*[1,2], Martina Kovarova[3], Clinton Jones[2], Daijha J. Copeland[2], Roopali Shrivastava[1], Michael D. Swanson[3], Craig Sykes[4], Phong T. Ho[3], Mackenzie L. Cottrell[3], Anush Sridharan[1], Samantha M. Fix[2], Orrin Thayer[3], Julie M. Long[3], Daria J. Hazuda[5], Paul A. Dayton[1], Russell J. Mumper[6], Angela D. M. Kashuba[4], J. Victor Garcia*[3]

Materials. 50:50 Poly(DL-lactide-*co*-glycolide) was purchased from LACTEL (Birmingham, AL; Cat. No. B6010-1P, Lot# 1614-09-01, weight average MW (Mw) 27.2 kDa, intrinsic viscosity (i.v.) 0.39, polydispersity index (PDI) 1.81). *N*-methyl-2-pyrrolidone (NMP, <USP>) was received from ASHLAND (Wilmington, DE, Product Code 851263, 100% NMP). Dolutegravir (DTG), Darunavir (DRV), Rilpivirine (RPV), Ritonavir (RTV), Atazanavir (ATV), Maraviroc (MVC), Zidovudine (AZT), Lamivudine (3TC), Stavudine (d4T), Emtricitabine (FTC), Tenofovir disoproxil fumarate (TDF), Etravirine (ETV), Efavirenz (EFV), Nevirapine (NVP), Raltegravir (RAL) were purchased from Selleckchem (Houston, TX; S2667-DTG, S7303-RPV, S1704-FTC). MK-2048 was generously provided by Dr. Daria Hazuda at Merck Research Laboratories (West Point, PA). Solutol-HS 15, phosphate buffered saline (0.01M PBS, pH 7.4), HPLC grade Acetonitrile were purchased from Sigma Aldrich (St. Louis, MO).

Supplementary Table 1. Chemical properties of ARV drugs.

**Drug**	**Chemical Structure*, MW (g/mol)**	**Solubility§ in NMP (mg/mL)**	**LogP†**	**pKa**	**IC90 (ng/mL)**	**Class**
MK-2048	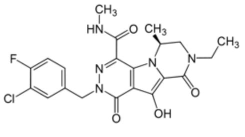 461.87	715 ± 5	2.67	9.79	33	ISTI
Darunavir (DRV)	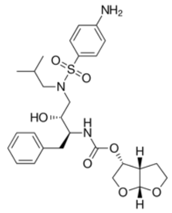 547.66	511 ± 2	1.8	11.4	2.43	PI
Ritonavir (RTV)	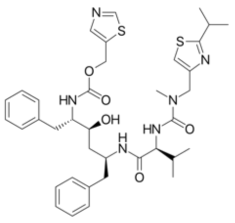 720.95	506 ± 6	3.9	2.8	2100	booster
Atazanavir (ATV)	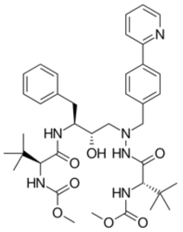 704.86	328 ± 2	4.5	4.3	14	PI
Dolutegravir (DTG)	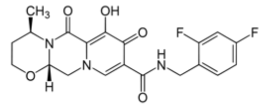 419.38	255 ± 4	2.2	8.2	64	ISTI
Rilpivirine (RPV)	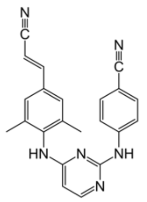 366.42	228 ± 5	4.86	5.6	12.5	NNRTI
Lamivudine (3TC)		250 ± 3	-1.4	4.08	17.43	NRTI
Stavudine (d4T)		248 ± 3	-0.7	10.26	30-300	NRTI
Emtricitabine (FTC)		202 ± 3	1.4	2.65	51	NRTI

Tenofovir Disoproxil Fumarate (TDF)	156 ± 2	1.25	3.75	5.54	NRTI
Etravirine (ETV)	122 ± 2	5.54	3.75	1.3	NNRTI
Efavirenz (EFV)	118 ± 2	4.6	10.2	0.2-2.2	NNRTI
Nevirapine (NVP)	57 ± 2	2.5	2.8	40	NNRTI
Raltegravir (RAL)	54 ± 2	-0.39	6.3	0.53	ISTI

*Chemical structures of drugs formulated in the ISFI and tested in vivo for PK.
†Log of drug partition coefficient (LogP) values were obtained from drugbank.ca
§Saturation solubility in *N*-methyl-2-pyrrolidone (NMP) quantified by HPLC analysis (n=3).
ISTI - Integrase Strand Transfer Inhibitor
PI - Protease Inhibitor
EI - Entry Inhibitor
NRTI - Nucleotide Reverse Transcriptase Inhibitor
NNRTI - Non-nucleotide Reverse Transcriptase Inhibitor

Supplementary Table 2. Stability of MK-2048 and DTG ISFI formulations.

Time (days)	25ºC ± 2ºC	25ºC ± 2ºC	40°C ± 2ºC/75%RH	40°C ± 2ºC/75%RH	5ºC ± 2ºC	5ºC ± 2ºC
	Residual MK-2048 (%)	Residual DTG (%)	Residual MK-2048 (%)	Residual DTG (%)	Residual MK-2048 (%)	Residual DTG (%)
0	100	100	100	100	100	100
7	100.8 ± 1.1	98.8 ± 1.1	99.1 ± 1.3	97.6 ± 2.1	100.2 ± 1.3	99.2 ± 0.8
14	99.9 ± 1.3	98.2 ± 0.9	98.2 ± 0.9	97.2 ± 1.4	99.1 ± 1.5	98.6 ± 0.5
30	99.4 ± 0.6	98.4 ± 1.6	98.5 ± 1.1	96.9 ± 1.7	98.5 ± 1.6*	97.8 ± 1.1*
90	98.7 ± 2.2	97.2 ± 1.2	97.2 ± 1.6	95.2 ± 1.8	-	-
120	97.2 ± 1.5	95.7 ± 1.4	95.7 ± 1.2	91.4 ± 2.2	-	-

* Formulation turned turbid at day 30, which was marked as last day of stability at 5ºC.
Sample (n=3) were analyzed by HPLC for residual drug concentration.

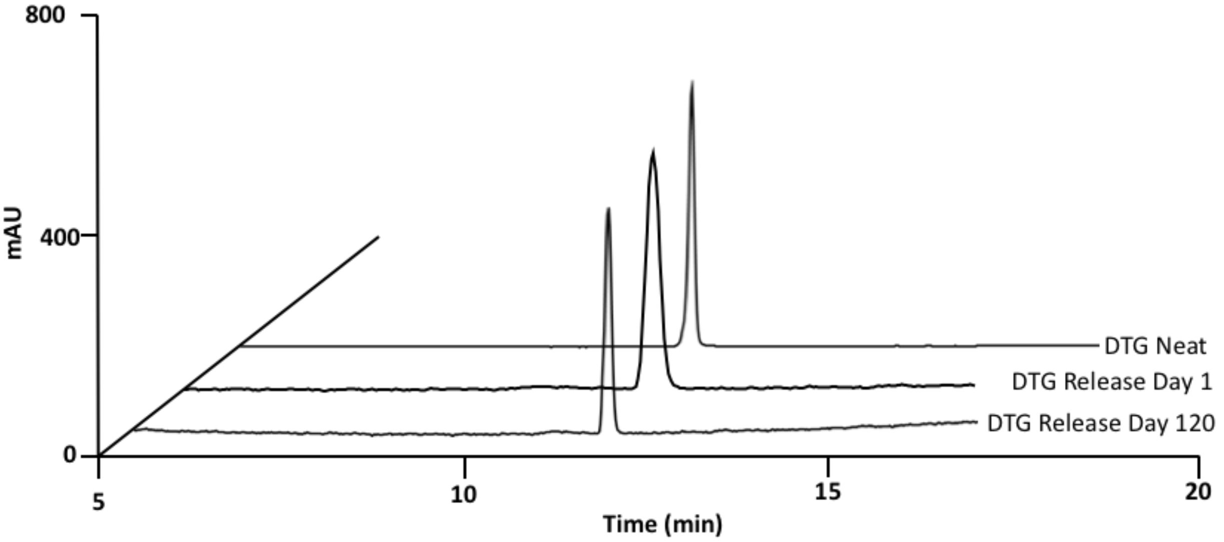

Supplementary Figure 1. Stability of DTG in the ISFI formulation during in vitro release. HPLC chromatograms of DTG (neat) and DTG released from an ISFI after day 1 and day 120 of incubations in PBS (pH 7.4) at 37ºC. DTG peak retained its shape and retention time with no degradation peaks detected over time in release samples.

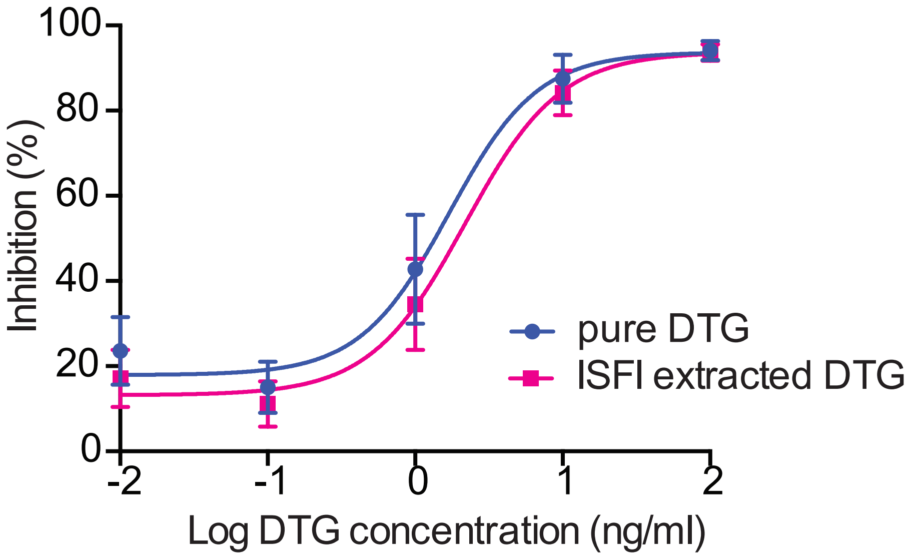

Supplementary Figure 2. Activity of DTG after 6 months in the ISFI formulation. DTG-ISFI was stored for 6 months in 25°C. The formulation was solidified in PBS and activity of DTG eluted from the solidified implant was evaluated as an ability to inhibit HIV infection in TZM-bl cells. Nonlinear fit of inhibitory activity of 1:10 dilutions of DTG is shown (red), compared to dilution of pure DTG (blue). Error bars indicated 95% confidence interval. Activity was measured in 3 independent experiments.

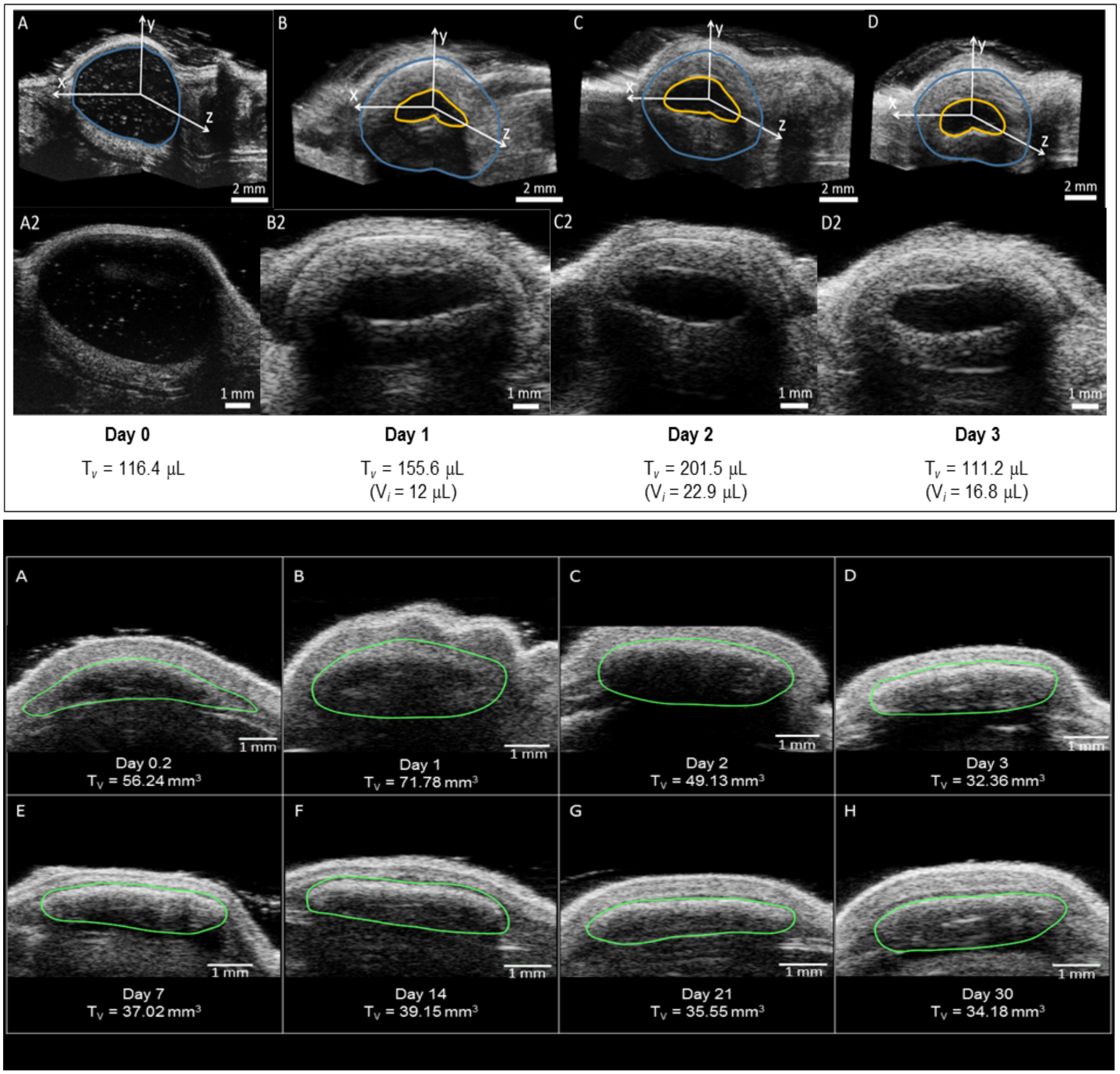

Supplementary Figure 3. Ultrasound (US) images of the ISFI after subcutaneous injection. ISFI formulations containing 1:2 or 1:8 w/w PLGA/NMP were injected (65 μL) s.c. into nude (nu/nu) mice (n = 7) and imaged using volumetric ultrasound for 30 days. **Top:** Placebo ISFI formulation (PLGA:NMP 1:2 w/w) imaged using volumetric ultrasound for three days. Images illustrate 3D sample volumes (A, B, C, D) and 2D slices through the same sample volumes (A2, B2, C2, D2) at days 0-3. T_v, measured volume of the injection site by 3D US Imaging depicted by the blue line. V_i, volume of internal liquid remaining (presumably, NMP solvent) depicted by the

orange line. Volume measurements are derived from the entire 3D data set in each case. Following injection, presence of a large pore and increased volume indicated that as residual NMP diffused out, influx of fluids led to swelling of the implant. At days 1-3 post injection, the swelling and the central pore are reduced and hardening of the shell is observed from the outside to the inside with a corresponding increase in 'white' contrast caused by the precipitated PLGA. **Bottom**: Images (A-H) illustrate 2D ultrasound scans of the largest cross-sectional area in the transverse imaging plane of placebo ISFI formulation (PLGA:NMP 1:2 w/w) over 30 days. T_V is the measured volume of the injection site based on the largest cross-sectional measurements obtained using both the transverse and sagittal planes. The average percent (%) volume decrease for placebo ISFI (n = 7) and DTG ISFI were 39% and 48% respectively at day 30.

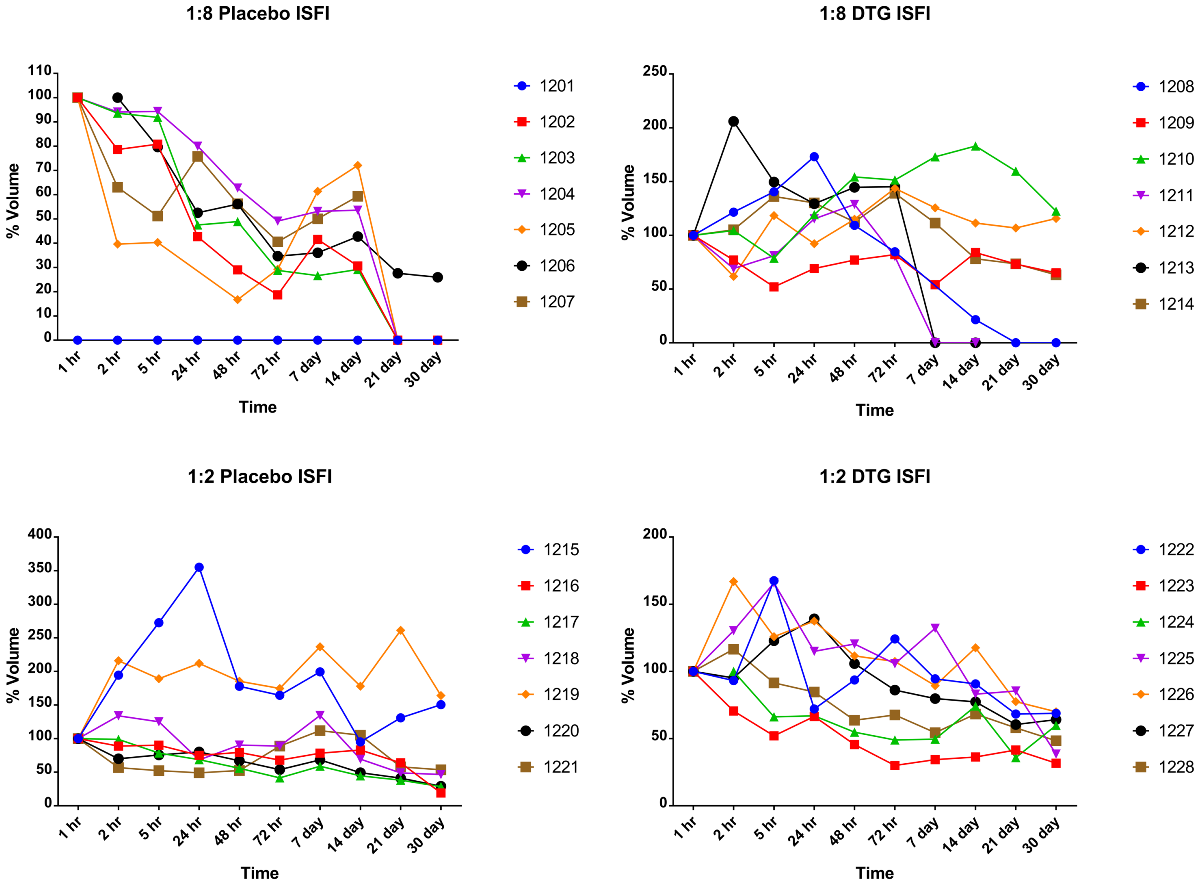

Supplementary Figure 4. Measurement of implant volume decrease over 30 days calculated from 2D in vivo ultrasound images for four different ISFI formulations plotted for individual mice in each group (n = 7 mice per group). **Top panel**: Placebo ISFI (PLGA/NMP 1:8 w/w) and DTG-ISFI (PLGA/NMP 1:8 w/w, [DTG] = 100 mg/mL); Bottom panel: Placebo ISFI (PLGA/NMP 1:2 w/w) and DTG-ISFI (PLGA/NMP 1:2 w/w, [DTG] = 100 mg/mL). Change in implant size (volume) is plotted as a percent (%) of the initial implant volume at time 1 h.

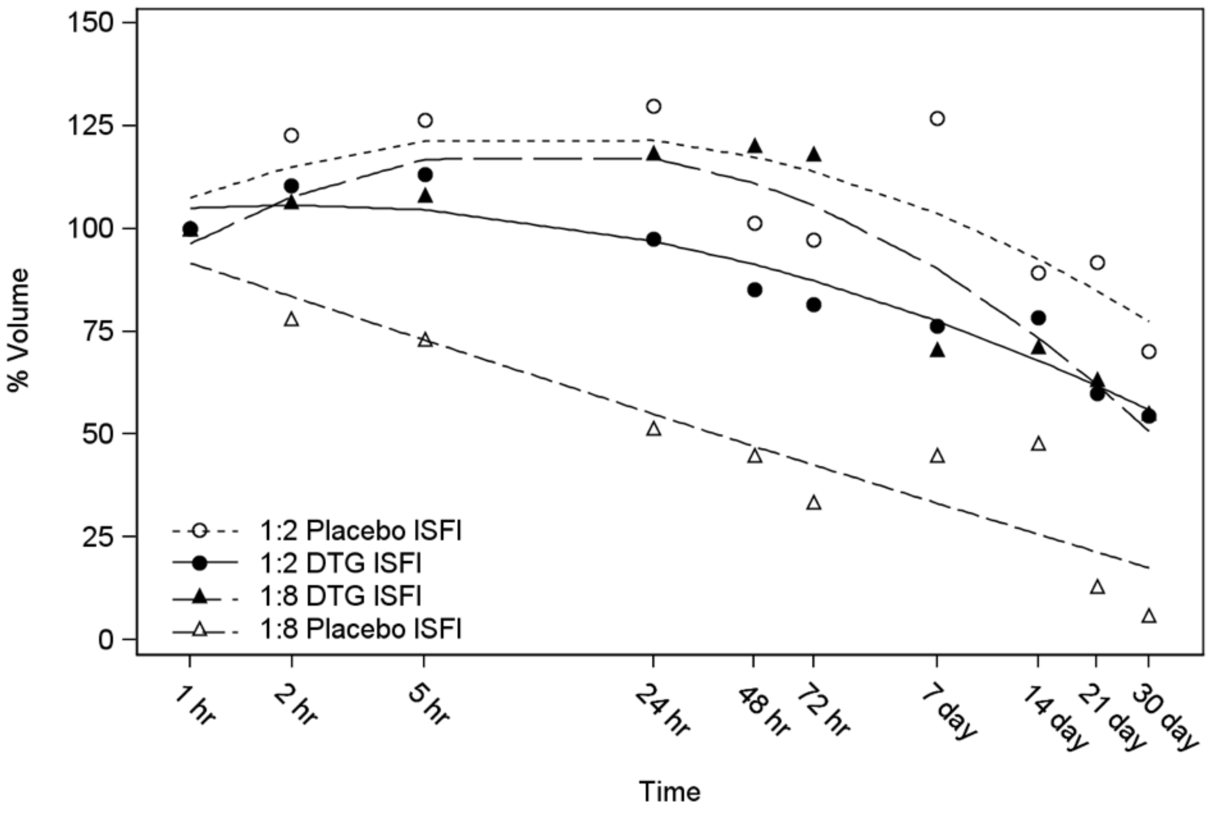

Supplementary Figure 5. Plot of raw (symbols) and model-estimated (lines) mean % volume of implant, by group and time. The mean % volume for the 1:8 placebo ISFI group is significantly lower than that of the other groups at day 1 ($p < 0.02$), and remained significantly lower than that of the 1:2 placebo ISFI group at day 30 ($p=0.03$); however, there were no significant differences across any of the groups for change from day 1 to day 30 (all p-values > 0.30, SAS modeling test).

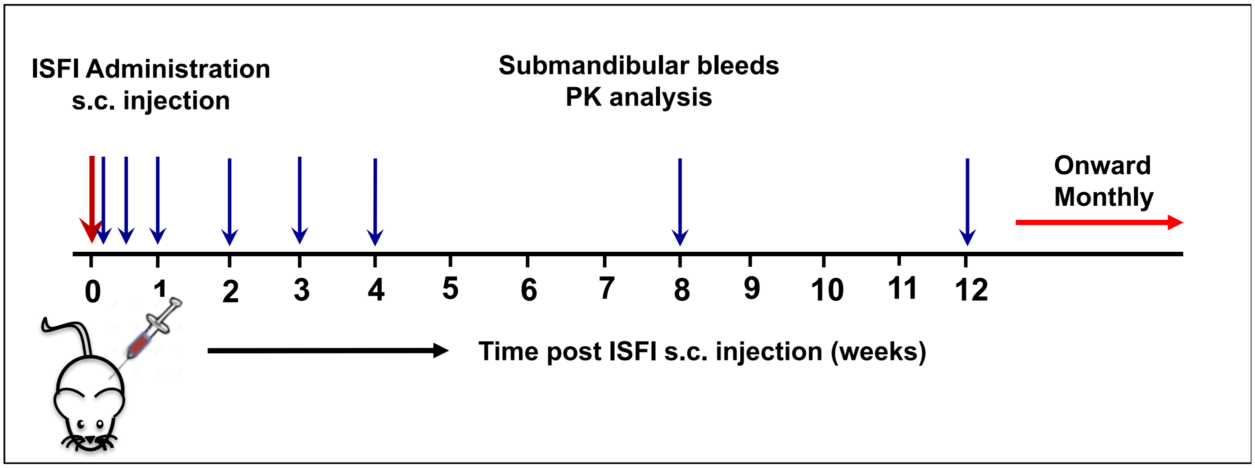

Supplementary Figure 6. Experimental Design. NSG mice were used to conduct in vivo pharmacokinetic studies. Mice (n=4) were injected with drug ISFI formulations subcutaneously and peripheral blood samples were collected longitudinally weekly post ISFI administration for the first 30 days and monthly for the following weeks until the end of each study. Drug concentrations in the plasma were quantified by LC-MS/MS analysis.
