# Supplementary material for: Ultra-long-acting tunable biodegradable and removable controlled release implants for drug delivery
Source: Nat Commun. 2019 Sep 20;10:4324. doi: 10.1038/s41467-019-12141-5 (PMC6754500; doi:10.1038/s41467-019-12141-5)
Supplement: Supplementary file 3 — Source Data File [file 41467_2019_12141_MOESM3_ESM.pdf]

Figure 4 (Left); MK-2048 ISFIs

	1:30 PLGA/NMP (w/w)		1:9 PLGA/NMP (w/w)		1:4 PLGA/NMP (w/w)		1:2 PLGA/NMP (w/w)	
Time (days)	%MK-2048 Released	SD (n=3)	%MK-2048 Released	SD (n=3)	%MK-2048 Released	SD (n=3)	%MK-2048 Released	SD (n=3)
0.002083333	0	0	30.9163516	1.092159642	0	0	1.870636861	0.253327073
0.020833333	30.46748096	1.670256004	35.07008202	4.05204971	3.520208274	0.98505586	3.306948655	0.377768814
0.720833333	70.9676166	1.130160876	41.79950159	0.889420624	5.70485145	1.060647873	10.37127018	2.25607737
2	92.15615947	4.730524957	54.01616795	0.167446292	14.23418161	1.093493411	15.58995949	1.7134228
3	95.47125711	1.017646396	63.60869795	1.012102979	31.46732969	2.1154821	19.44036061	0.943874576
5	103.4665063	1.621818869	71.70750615	1.972497	43.08530731	3.010522569	24.17246855	1.336580382
7			81.47616241	4.050710155	51.89744576	3.16732921	26.55192177	1.61054933
9			85.66511006	1.182865647	60.857445	3.24871308	31.77334588	4.67037844
15			86.46047551	0.998501825	82.14662625	1.373777375	38.10804353	1.569226939
20					85.99548762	3.2187364	41.37427082	3.4192154
27					90.14107881	1.452094584	50.18352767	6.2319861
35					96.22657724	2.796524511	57.860903	1.34188441
43					107.5135102	3.138510238	61.78507357	3.24440617
55							67.56421307	4.71263956
77							73.74912975	3.23875345
96							79.07482977	3.38296943
125							81.63633192	6.62331064
166							95.25782192	5.63222965
208							104.5215146	5.16358552

Figure 4 (Right); DTG ISFIs

	1:16 PLGA/NMP (w/w)		1:8 PLGA/NMP (w/w)		1:4 PLGA/NMP (w/w)		1:2 PLGA/NMP (w/w)	
Time (days)	%DTG Released	SD (n=3)	%DTG Released	SD (n=3)	%DTG Released	SD (n=3)	%DTG Released	SD (n=3)
0	17.02361207	5.038111072	3.671179156	0.506550004	8.557123625	0.153497409	1.386104176	0.347033761
0.02	47.30226675	9.365623484	12.23917201	1.00624262	13.43082076	0.138930631	3.38421047	0.138564534
0.04	58.20737459	8.290337537	17.22392683	1.281635436	17.56271347	0.059278916	7.465338591	0.481868262
0.08	69.75707093	10.536765	21.59597575	0.948948887	21.9037034	0.592642267	10.36516016	0.605503327
0.16	79.64927551	10.14525215	25.81614203	0.694707534	24.57498456	1.311160066	11.90769138	0.662437404
0.25	84.74223623	10.08061326	28.71927646	1.456110692	25.79254358	1.943463507	13.09639525	0.939462466
0.33	88.29941149	3.28636236	30.97209246	1.741524336	26.6705238	2.01756257	13.79983987	0.957297097
1	95.14280269	3.928059487	36.61853075	4.103286605	29.50984782	1.771993821	16.7666178	2.087117259
2	96.21959657	4.675743889	39.71984293	3.688151752	31.3870432	1.872010812	18.11340318	2.795048242
3	97.12437009	3.28636236	41.87473765	3.088597724	32.40980326	1.792647152	18.56763499	3.486782488
4	97.50885353	4.87630601	43.49708679	2.078022415	34.44138621	1.145140292	18.71916076	3.824130168
5	98.37419993	5.948287373	44.99284313	1.49845752	35.35016361	2.279680839	18.49723977	3.581725282
6	99.63999601	3.883860268	46.86801243	1.497710172	35.38655891	1.567541157	18.91967283	3.795925146
7	100.5659731	4.523165083	48.85469581	1.446263039	34.97370587	0.609894221	18.38333885	3.43650479
9			52.62044299	1.943441925	36.92166182	2.398761047	18.17681184	3.421981498
12			56.93800164	2.506795824	36.71790223	2.046433735	17.68766633	3.23996431
14			60.82235677	3.018134809	36.62046326	1.033843165	18.12931557	3.389693525
21			70.15791286	3.444127795	37.16641581	2.198164118	17.32630103	2.601577554
28			70.19836858	1.91276227	38.65327046	0.83802002	19.84985026	2.644374394
35			76.96665446	2.695505909	38.2244788	1.448971183	19.95595114	2.550852453
42			80.28036295	2.877283847	42.09072228	1.325046731	21.7278358	2.369282853
49			82.93567133	2.940684378	44.46628336	1.077048402	21.86390754	0.6686399
56			84.92774273	2.569146605	45.61534643	0.409249908	22.22366182	0.773560632
63			85.11420106	1.856735044	45.40116259	2.232907808	23.81086322	1.560763252
70			87.27746795	1.764390848	46.60586024	0.486876207	24.67857553	2.058989857
77			91.6329768	1.13796789	50.6781927	1.07896242	25.7567296	1.77831783
84			92.1672809	1.46782234	53.2796782	1.96757789	27.3479829	2.06768298
91			93.86818645	1.96789032	54.5638902	1.17896782	28.6598531	1.34792913
98			96.1389129	2.06784003	55.7856179	1.86789026	30.2527927	1.75677686
105			97.3789027	1.78567589	57.4678491	2.86852353	32.0347892	0.97857861
112			98.5246743	1.46879824	58.5547892	1.04678289	33.6497567	1.47878971
120			99.2789404	1.15687942	60.27942789	2.28975892	34.0367821	2.78958178

Source Data for Figure 6

	Time point (days)	1:8 (PLGA/NMP) Placebo ISFI				1:8 (PLGA/NMP) DTG ISFI				1:2 (PLGA/NMP) Placebo ISFI						1:2 (PLGA/NMP) DTG ISFI				
1 hr	0.04166667	0.0455886	0.0316277	0.0179869	0.0376251	0.1068586	0.05218976	0.0368515	0.0521174	0.0199134	0.0823509	0.0496345	0.0262997	0.0816421	0.0411082	0.0550508	0.0304234	0.0240423	0.038665	0.0436343
2 hr	0.08333334	0.0302084	0.0255632	0.0290743	0.0246975	0.0897909	0.05057496	0.0235801	0.0539639	0.0351116	0.0725006	0.0446519	0.0434052	0.0489341	0.0217915	0.0368965	0.0349493	0.0395684	0.0409582	0.0397008
5 hr	0.2083333	0.031865	0.023803	0.026943	0.020204	0.057679	0.046258	0.045935	0.070707	0.046449	0.062105	0.041669	0.034071	0.053573	0.021457	0.025383	0.036797	0.029155	0.047738	0.032541
24 hr	1	0.0175983	0.0140851	0.0219762	0.0265977	0.07503489	0.06084852	0.0409307	0.0578935	0.05128	0.0481339	0.0369852	0.0497873	0.0600699	0.0198317	0.0309148	0.0304688	0.0367698	0.057906	0.0286079
48 hr	2	0.017331	0.0131631	0.0179825	0.0209768	0.0522108	0.03652911	0.0221874	0.0221776	0.0207113	0.0296035	0.0181473	0.0352339	0.0269733	0.0122944	0.0121615	0	0.0225085	0.038976	0.0110346
72 hr	3	0.0075	0.0066	0.0196	0.0154	0.0454	0.0539	0.0232	0.0652	0.0212	0.0216	0.0135	0.0183	0.0057	0.0135	0.0123	0.0154	0.0178	0.0339	0.0119
7 day	7	0.015	0.0097	0.0187	0.0203	0.0658	0.0476	0.0244	0	0	0.0286	0.0164	0.031	0.0147	0.0195	0.0118	0.0117	0.016	0.0252	0.0095
14 day	14	0.0111	0.0075	0.0126	0.0213	0.0559	0.0501	0.0259	0.0585	0.0155	0.0287	0.0148	0.0291	0.0281	0.0258	0.0172	0.0204	0.0142	0.0141	0.016
21 day	21	0.0110754	0.0074565	0.012579	0.0212809	0.05184829	0.05422302	0.0479642	0	0.0138585	0.0287425	0.0120878	0.0261947	0.028034	0.01164	0.0132477	0.0148292	0.0159971	0.0212704	0.0194855
30 day	30	0.0110754	0.0074565	0.012579	0	0.06863852	0.06748521	0.0433444	0.0324518	0.0177673	0.0107337	0.013238	0.0271667	0.0198824	0.0189406	0.0178974	0.0099114	0.0225933	0.029468	0.0208268

Figure 7A

days after administration	Dolutegravir Dose (250 mg/kg)							
	Plasma Dolutegravir (ng/ml)							
1	8260	11300	9230	6970				
3	5290	3970	5530	4330				
7	7480	3910	3950	4510	6050	3440	4250	5900
14	3630	1710	2880	4210	4010	3840	3170	4200
21					3720	2620	3310	4150
28	2630	1020	1570		3940	1710	2750	2460

days after administration	Rilpivirine Dose (100 mg/kg)							
	Plasma Rilpivirine (ng/ml)							
1	135	305	193	263				
3	54.9	66.9	54.6	102				
7	48.8	20.7	24.3	46.3				
14	47	23.5	37.8	53.7				
21	49.2	32.8	92.9	92				
28	69.7	42.6	43.8	107				

days after administration	Darunavir Dose (300 mg/kg)							
	Plasma Darunavir (ng/ml)							
1	368	284	308	281				
3	149	74.1	83.5	68.2				
7	29.2	40	44.1	34.6				
14	18.2	7.43	15.7	13.3				
21	8.29	3.29	16	13				
28	6.37	5.44	10.2	8.3				

days after administration	Atazanavir Dose (300 mg/kg)							
	Plasma Atazanavir (ng/ml)							
1	362	508	300	375				
3	23.3	52	64.1	70.7				
7	13.7	30.8	18.6	20.6				
14	11.1	10.5	7.7	8.02				
21	10.1	7.49	8.14	6.58				
28	11.5	7.07	9.34	7.83				

days after administration	Ritonavir Dose (33.8mg/kg)							
	Plasma Ritonavir (ng/ml)							
1	51.3	40.9	32.2	44.8				
3	10	10.7	13.2	17.6				
7	7.37	11.1	15.1	14.5				
14	4.37	4.75	5.46	7.62				
21	2.64	2.57	5.02	4.77				
28	1.87	1.36	3.74	3.4				

days after administration	MK2048 Dose (550mg/kg)							
	Plasma MK2048 (ng/ml)							
1	1580	1240	1110	1160				
3	602	352	342	619				
7	363	196	299	322				
14	285	189	208	315				
21	283	178	213	268				
28	165	135	152	187				

Figure 7B

days after administration	Dolutegravir Dose (250 mg/kg)							
	Plasma Dolutegravir (ng/ml)							
1	8260	11300	9230	6970				
3	5290	3970	5530	4330				
7	7480	3910	3950	4510	6050	3440	4250	5900
14	3630	1710	2880	4210	4010	3840	3170	4200
21					3720	2620	3310	4150
28	2630	1020	1570		3940	1710	2750	2460

days after administration	Dolutegravir Dose (100 mg/kg)							
	Plasma Dolutegravir (ng/ml)							
1	4930	6440	5730	6090	5320			
3	2470	3910	3270	4990	5140			
7	1330	1640	1580	3000	2560			
14	1270	1620	1350	3050	1960			
21	1180	1240	916	2310	1460			
28	817	803	502					

days after administration	Darunavir Dose (300 mg/kg)							
	Plasma Darunavir (ng/ml)							
1	368	284	308	281				
3	149	74.1	83.5	68.2				
7	29.2	40	44.1	34.6				
14	18.2	7.43	15.7	13.3				
21	8.29	3.29	16	13				
28	6.37	5.44	10.2	8.3				

days after administration	Darunavir Dose (100 mg/kg)							
	Plasma Darunavir (ng/ml)							
1	119	121	99.4	136	173			
3	31.7	44.4	30.7	92.2	67.3			
7	15	19.1	14.6	41.4	31.6			
14	7.95	11.6	8.46	15.8	15			
21	11.3	9.14	7.12	15	9.68			
28	5.66	6.26	3.04					

days after administration	Ritonavir Dose (33.8mg/kg)							
	Plasma Ritonavir (ng/ml)							
1	51.3	40.9	32.2	44.8				
3	10	10.7	13.2	17.6				
7	7.37	11.1	15.1	14.5				
14	4.37	4.75	5.46	7.62				
21	2.64	2.57	5.02	4.77				
28	1.87	1.36	3.74	3.4				

days after administration	Ritonavir Dose (11.4 mg/kg)							
	Plasma Ritonavir (ng/ml)							
1	6.12	7.46	5.35	10.8	7.91			
3	2.62	3.45	2.95	9.2	5.54			
7	1.38	1.87	1.85	4.68	3.25			
14	1.09	1.29	1.21	2.07	2.17			
21	1.07	1	1.09	2.3	1.03			
28	1	1	1	1	1			

Raw data for Figure 9B

Time after removal (d)	Plasma DTG in ng/ml				
0	611	648	529	822	432
1	132	16.8	5.42	17.8	12.9
3	27.6	2.49	1	1.22	1.34
7	8	1	1	1	1
13	1.03	1	1	1	1
21	1	1	1	1	1

Raw data for Figure 9c

Time after removal (d)	Plasma DTG in ng/ml				
0	100	100	100	100	100
1	21.6039	2.59259	1.02458	2.16545	2.98611
3	4.51718	0.38426	0.18904	0.14842	0.31019
7	1.30933	0.15432	0.18904	0.12165	0.23148
13	0.16367	0.15432	0.18904	0.12165	0.23148
21	0.16367	0.15432	0.18904	0.12165	0.23148

Source Data for Figure S2

**DTG concentration (ng/ml)**	Pure DTG												ISFI extracted DTG											
100	90.6988	89.8536	90.2681	87.2532	97.0061	97.0613	97.3212	96.3394	96.4594	95.8463	95.9251	95.7592	89.2506	90.4512	90.1174	88.6404	97.0155	96.0687	95.9044	97.0114	95.1574	94.8311	94.9239	94.0212
10	77.3096	72.8052	79.1903	74.293	94.8768	95.5138	94.5387	93.7508	94.4993	92.4801	91.4255	89.1954	73.6738	74.6734	74.8493	73.4926	94.0108	93.367	94.3515	91.6404	81.6362	85.5311	87.5165	85.1458
1	21.5578	23.5608	23.279	17.7787	60.862	60.2236	50.1064	52.4216	51.4357	46.9083	44.9225	51.4469	16.9047	17.5049	8.14667	9.84854	55.7859	50.2505	47.4546	45.7751	37.6276	38.2829	47.4395	48.6678
0.1	23.7492	13.3497	10.4802	9.61704	23.9515	19.2485	20.3232	6.0431	10.6584	10.547	23.8196	21.9586	10.2272	0.43966	1.29548	15.8244	13.2472	18.4727	11.5246	8.81078	6.89076	8.81071	27.6808	7.3596
0.01	17.034	11.9607	20.2588	12.325	33.6391	21.2525	18.6451	4.97913	49.1381	39.3937	27.242	26.8652	8.72514	9.13969	16.7432	34.4645	19.938	4.48216	18.3286	2.53469	23.409	10.7897	26.2437	24.7251
0.001	17.3318	5.16832	10.875	8.28368									12.7198	13.5525	2.75644	13.0572								

**Log DTG concentration (ng/ml)**	Pure DTG												ISFI extracted DTG											
2	90.6988	89.8536	90.2681	87.2532	97.0061	97.0613	97.3212	96.3394	96.4594	95.8463	95.9251	95.7592	89.2506	90.4512	90.1174	88.6404	97.0155	96.0687	95.9044	97.0114	95.1574	94.8311	94.9239	94.0212
1	77.3096	72.8052	79.1903	74.293	94.8768	95.5138	94.5387	93.7508	94.4993	92.4801	91.4255	89.1954	73.6738	74.6734	74.8493	73.4926	94.0108	93.367	94.3515	91.6404	81.6362	85.5311	87.5165	85.1458
0	21.5578	23.5608	23.279	17.7787	60.862	60.2236	50.1064	52.4216	51.4357	46.9083	44.9225	51.4469	16.9047	17.5049	8.14667	9.84854	55.7859	50.2505	47.4546	45.7751	37.6276	38.2829	47.4395	48.6678
-1	23.7492	13.3497	10.4802	9.61704	23.9515	19.2485	20.3232	6.0431	10.6584	10.547	23.8196	21.9586	10.2272	0.43966	1.29548	15.8244	13.2472	18.4727	11.5246	8.81078	6.89076	8.81071	27.6808	7.3596
-2	17.034	11.9607	20.2588	12.325	33.6391	21.2525	18.6451	4.97913	49.1381	39.3937	27.242	26.8652	8.72514	9.13969	16.7432	34.4645	19.938	4.48216	18.3286	2.53469	23.409	10.7897	26.2437	24.7251
-3	17.3318	5.16832	10.875	8.28368									12.7198	13.5525	2.75644	13.0572								
